# Deciphering decadal urban ozone trends from historical records since 1980

**DOI:** 10.1093/nsr/nwae369

**Published:** 2024-10-24

**Authors:** Haolin Wang, Xiao Lu, Paul I Palmer, Lin Zhang, Keding Lu, Ke Li, Tatsuya Nagashima, Ja-Ho Koo, Hiroshi Tanimoto, Haichao Wang, Meng Gao, Cheng He, Kai Wu, Shaojia Fan, Yuanhang Zhang

**Affiliations:** School of Atmospheric Sciences, Sun Yat-sen University, and Key Laboratory of Tropical Atmosphere-Ocean System, Ministry of Education, Zhuhai 519082, China; Guangdong Provincial Observation and Research Station for Climate Environment and Air Quality Change in the Pearl River Estuary, Southern Marine Science and Engineering Guangdong Laboratory (Zhuhai), Zhuhai 519082, China; School of GeoSciences, University of Edinburgh, Edinburgh EH9 3FF, UK; School of Atmospheric Sciences, Sun Yat-sen University, and Key Laboratory of Tropical Atmosphere-Ocean System, Ministry of Education, Zhuhai 519082, China; Guangdong Provincial Observation and Research Station for Climate Environment and Air Quality Change in the Pearl River Estuary, Southern Marine Science and Engineering Guangdong Laboratory (Zhuhai), Zhuhai 519082, China; School of GeoSciences, University of Edinburgh, Edinburgh EH9 3FF, UK; National Centre for Earth Observation, University of Edinburgh, Edinburgh EH9 3FF, UK; Laboratory for Climate and Ocean-Atmosphere Studies, Department of Atmospheric and Oceanic Sciences, School of Physics, Peking University, Beijing 100871, China; State Key Joint Laboratory of Environmental Simulation and Pollution Control, The State Environmental Protection Key Laboratory of Atmospheric Ozone Pollution Control, College of Environmental Sciences and Engineering, Peking University, Beijing 100871, China; Jiangsu Key Laboratory of Atmospheric Environment Monitoring and Pollution Control, Collaborative Innovation Center of Atmospheric Environment and Equipment Technology, School of Environmental Science and Engineering, Nanjing University of Information Science & Technology, Nanjing 210044, China; National Institute for Environmental Studies, Tsukuba 305-8506, Japan; Department of Atmospheric Sciences, Yonsei University, Seoul 03722, South Korea; National Institute for Environmental Studies, Tsukuba 305-8506, Japan; School of Atmospheric Sciences, Sun Yat-sen University, and Key Laboratory of Tropical Atmosphere-Ocean System, Ministry of Education, Zhuhai 519082, China; Guangdong Provincial Observation and Research Station for Climate Environment and Air Quality Change in the Pearl River Estuary, Southern Marine Science and Engineering Guangdong Laboratory (Zhuhai), Zhuhai 519082, China; Department of Geography, Hong Kong Baptist University, Hong Kong, China; School of Atmospheric Sciences, Sun Yat-sen University, and Key Laboratory of Tropical Atmosphere-Ocean System, Ministry of Education, Zhuhai 519082, China; Guangdong Provincial Observation and Research Station for Climate Environment and Air Quality Change in the Pearl River Estuary, Southern Marine Science and Engineering Guangdong Laboratory (Zhuhai), Zhuhai 519082, China; Department of Civil and Environmental Engineering, University of California, Irvine, CA 92697, USA; School of Atmospheric Sciences, Sun Yat-sen University, and Key Laboratory of Tropical Atmosphere-Ocean System, Ministry of Education, Zhuhai 519082, China; Guangdong Provincial Observation and Research Station for Climate Environment and Air Quality Change in the Pearl River Estuary, Southern Marine Science and Engineering Guangdong Laboratory (Zhuhai), Zhuhai 519082, China; State Key Joint Laboratory of Environmental Simulation and Pollution Control, The State Environmental Protection Key Laboratory of Atmospheric Ozone Pollution Control, College of Environmental Sciences and Engineering, Peking University, Beijing 100871, China

**Keywords:** air quality, surface ozone, trends

## Abstract

Ozone pollution is a major environmental threat to human health. Timely assessment of ozone trends is crucial for informing environmental policy. Here we show that for the most recent decade (2013–2022) in the northern hemisphere, warm-season (April–September) mean daily 8-h average maximum ozone increases much faster in urban regions with top ozone levels (mainly in the North China Plain, 1.2 ± 1.3 ppbv year^−1^) than in other, low-ozone regions (0.2 ± 0.9 ppbv year^−1^). These trends widen the ozone differences across urban regions, and increase extreme pollution levels and health threats from a global perspective. Comparison of historical trends in different urban regions reveals that ozone increases in China during 2013–2022 differ in magnitude and mechanisms to historical periods in other regions since 1980. This reflects a unique chemical environment characterized by exceptionally high nitrogen oxides and aerosol concentrations, where reducing ozone precursor emissions leads to substantial ozone increase. Ozone increase in China has slowed down in 2018–2022 compared to 2013–2017, driven by ongoing emission reductions, but with ozone-favorable weather conditions. Historical ozone evolution in Japan and South Korea indicates that ozone increases should be suppressed with continuous emission reduction. Increasing temperature and associated wildfires have also reversed ozone decreases in the USA and Europe, with anthropogenic ozone control slowing down in recent decades.

## Teaser

The historical evolution of surface ozone levels across the globe encapsulates crucial insights into the underlying drivers and regulatory pathways of ozone variability.

## INTRODUCTION

Ambient ozone is a key environmental threat to human health and ecosystems [1–3]. It is formed from photochemistry involving carbon monoxide (CO), nitrogen oxides (NO*_x_* ≡ NO + NO_2_) and volatile organic compounds (VOCs). Ozone in the urban polluted boundary layer has an e-folding lifetime of hours to days, so its temporal trend is highly variable in space [4]. Analyses of urban ozone trends provide a timely assessment of how ozone exposure affects human health and the effectiveness of air quality mitigation measures.

The Tropospheric Ozone Assessment Report Phase I (TOAR I) provided the first global overview of surface ozone trends from 1970 to 2014 [5,6]. It revealed that urban ozone levels generally decreased across Europe and the USA during boreal summer and increased over developing regions. This result reflects an equatorward redistribution of anthropogenic emissions since 1980 [7,8]. Ozone monitoring networks located in rapidly developing regions, such as China and India, have emerged since 2013. These data provide new opportunities to examine the spatiotemporal shifts of the global ozone pollution pattern in the most recent decade of 2013–2022 [9] when anthropogenic emissions show dramatic changes in response to country-level policy-driven clean air actions and to the worldwide Coronavirus disease (COVID-19) pandemic [10,11]. Ozone trends in the USA and Europe after 2013 remain largely unexplored. Chinese anthropogenic NO*_x_* emissions peaked in 2012 and then decreased dramatically afterward [12], but surface ozone increased significantly in 2013–2019 [13,14]. The spread of COVID-19 led to a substantial decrease in anthropogenic emissions, but whether this has significantly affected the decadal surface ozone trend is unclear [15]. Previous studies have typically focused on long-term (∼20 years) ozone trends [16–18], but comparing regional ozone trends on a decadal timescale, which provides more granular information on ozone pollution stage and tendency, has not yet been addressed.

Here we provide an updated picture of ozone pollution in the urban agglomeration (including urban and suburban areas) across the northern hemisphere, including new observations from China and India, in the decade of 2013–2022. Our primary goal is to examine recent ozone trends in regions with different ozone pollution levels and their implications with regard to extreme ozone pollution and associated damage. We also compare regional ozone trends and the corresponding changes in emissions and climate during 2013–2022 with previous decades, since 1980. This allows us to achieve a historical perspective on the spatiotemporal evolution of decadal urban ozone trends, and to examine the track of ozone trends in response to mitigation strategies in different periods and regions.

## RESULTS AND DISCUSSION

### Distribution and trends of urban ozone pollution for the most recent decade (2013–2022)

**Figure 1.**
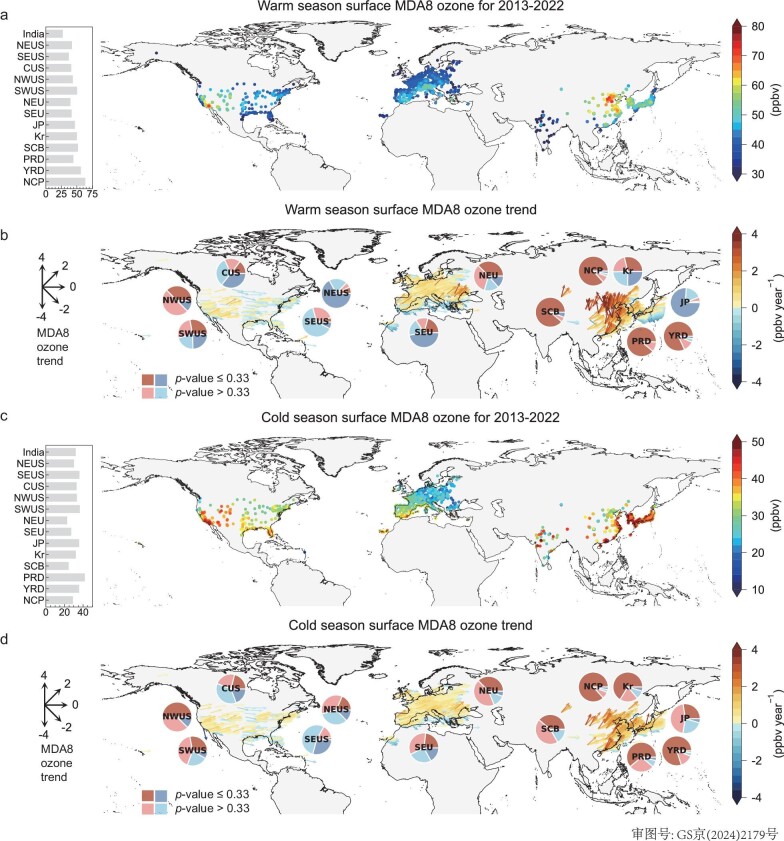
Mean daily 8-h average maximum (MDA8) ozone and trends in 2013–2022 in the warm (April–September) and cold (October–March) seasons. (a) and (c) show the mean MDA8 ozone level at each site for the warm and cold seasons, respectively. The bars inset show the site-average MDA8 ozone concentration in each region (defined in Fig. S1, with the number of sites in each region summarized in Table S1). (b) and (d) show the MDA8 ozone trend for 2013–2022 for the warm and cold seasons, respectively. Both directions and colors of the vectors indicate the MDA8 ozone trend in ppbv year^−1^. The pie charts inset summarize the percentage of sites with positive and negative trends for each region, separated by the *P*-values derived from the trend estimate.

Figure 1 presents the distribution of mean daily 8-h average maximum (MDA8) ozone and trends at urban and suburban sites across the northern hemisphere from 2013 to 2022 for the boreal warm (April–September) and cold (October–March) seasons. These ozone measurements are from national monitoring networks in China, India, South Korea, Japan, Europe and the USA (Materials and Methods), representing the most extensive coverage of urban ozone monitoring in the northern hemisphere so far. These monitoring networks explicitly categorize urban and suburban sites. Trends are derived from the generalized least-squares method with autocorrelation (Materials and Methods). Fig. S2 shows the time series of ozone for different regions. We focus on the MDA8 ozone, a standard metric for assessing policy-relevant ozone air quality in many countries, and in cohort studies examining the responses of human health to ozone exposure. Results for other ozone metrics, such as those for assessing cumulative health and vegetation exposure, are shown in the Supplementary Data.

Figure 1a shows that the North China Plain (NCP), Yangtze River Delta (YRD) and southern California in Southwest USA (SWUS) remain hotspots of ozone pollution in the most recent decade, 2013–2022. This pattern has not significantly changed since the last TOAR estimate and subsequent follow-up studies [5,19], which focused on the earlier periods of 2000–2014 or 2013–2017. The 2013–2022 mean MDA8 ozone in the NCP, YRD and SWUS is 63.7 ± 4.3 (mean ± standard deviation, number of sites (n) = 59), 56.3 ± 5.2 (n = 97) and 50.4 ± 9.6 ppbv (n = 108), respectively. These high ozone levels are driven by intensive local anthropogenic emissions and active photochemistry in the boreal summer [14,18,20,21]. The follow-up regions with high urban MDA8 ozone levels are the Sichuan Basin (SCB) in China, South Korea, Japan, the Intermountain West USA and the alpine region in Central Europe. The first three regions have experienced intense anthropogenic ozone production, while the latter two have high natural background ozone [22,23]. Previous studies of global ozone distribution have not included India due to the scarcity of observational sites. Here we show that the April–September mean MDA8 ozone for India sites is 27.2 ± 7.8 ppbv (n = 45) in 2018–2021. The relatively low ozone in India compared to other populated regions can be largely attributed to the Indian summer monsoon, which brings wet and cool weather conditions and strong upward transport, which are not favorable for ozone production and accumulation [24,25].

Figure 1b illustrates the large spatial heterogeneity in urban ozone trends across the northern hemisphere in the most recent decade. Warm-season MDA8 ozone in China has increased at a mean rate of 1.4 ± 1.2 ppbv year^−1^ (3.6% year^−1^) over 384 sites during 2013–2022, with over 92.7% sites showing positive trends (90.4% with *P*-value ≤ 0.33). The NCP region records the highest regional mean ozone increase of 1.8 ± 1.2 ppbv year^−1.^ The increase rate China-wide, and in the NCP region, is lower in 2013–2022 compared to the reported 2.4 and 3.3 ppbv year^−1^ in 2013–2019 using a consistent trend estimation method [13]. A previous study has reported a notable ozone decrease in eastern China in the warm season of 2020, which can be attributed to both the policy-driven emission reduction of ozone precursors and weather anomalies [26]. This ozone decrease propagates to 2021 (Fig. S2), but we find a strong rebound of surface ozone level in 2022 over all major city clusters, suggesting that ozone pollution in China is not yet under control. Metrics for accumulative human health and vegetation exposure show even larger relative increasing trends (∼6% year^−1^) compared to the MDA8 metric in China in 2013–2022 (Fig. S3).

Figure 1b also illustrates the contrasting warm-season ozone trends over the western and eastern USA. Over Northwest USA (NWUS) and SWUS (Table S3), 87.5% and 54.1% of sites show an increase in MDA8 ozone levels in 2013–2022, respectively, with the largest trend reaching 1.2 ppbv year^−1^ (2σ uncertainty [±1.0], *P*-value < 0.05) in San Bernardino, California. The last TOAR estimate reported a remarkable ozone decrease in urban California during 2000–2014 [5]. However, our analysis suggests that this decrease is reversed after 2013. In contrast, over Northeast USA (NEUS), southeast USA (SEUS) and Central USA (CUS), 87.1%, 70.7% and 67.9% of sites show an ozone decrease, with regional mean trends ranging from −0.3 to −0.5 ppbv year^−1^. South Korea has approximately half of the sites (50.4%) reporting positive ozone MDA8 trends during 2013–2022. Over 70% of the sites in Northern Europe exhibit ozone increases, whereas more than half of the sites in Southern Europe show a decrease. Most Japanese sites (94.0%) show an ozone decrease in 2013–2022, with a mean rate of −0.8 ± 0.5 ppbv year^−1^, after a long-term period since 1980 when ozone increased [27,28] (Fig. S2). Factors driving these trends will be discussed in the last section. Data coverage in India (4 years) is too short to determine a statistically robust trend estimate, but we find that ozone there has continually decreased since 2018 (Fig. S2).

We also examine the potential impact of the COVID-19 pandemic in 2020 on ozone trends. Previous studies reported a notable ozone increase in eastern China during the COVID-19 lockdown period in February 2020 [29,30]. In contrast to the observed short-term wintertime ozone increase, except for the Pearl River Delta (PRD), Fig. S2 shows a notable decrease in warm-season mean MDA8 ozone in 2020 over almost all regions. Keller *et al.* (2021) attributed the ozone decrease to a worldwide reduction in ozone precursors in 2020 [31–33]. We further show in Fig. S4 the influence of the 2020 data on regional ozone trends during 2013–2022. When including observations from 2020, we find 70% of the stations exhibit a considerably reduced trend, resulting in regional trends during 2013–2022 being smaller by −0.01 to −0.34 ppbv year^−1^. However, excluding the 2020 data does not change the sign of the 2013–2022 regional warm-season ozone trends.

Figure 1c and d show that the spatial patterns of ozone levels and trends are generally different for the cold and warm seasons. A significant difference is that more sites in the cold season than the warm season show increasing trends in MDA8 ozone and other ozone metrics, suggesting a gradual extension of ozone pollution into the cold season. This phenomenon is most evident in East Asia, where >70% of urban sites in China, Japan and South Korea [34] show significant increases of up to 3.5 ppbv year^−1^. This can be mostly attributed to elevated ozone production from the reduction of NO*_x_* emissions under the VOC-limited ozone production regime in the cold season [30]. Including the 2020 ozone observations changes the 2013–2022 cold-season ozone trend by −0.004 to −0.13 ppbv year^−1^ except for SWUS (0.03 ppbv year^−1^) and Japan (0.04 ppbv year^−1^).

### Growing differences in ozone pollution between heavy-ozone-polluted and light-ozone-polluted urban regions

**Figure 2.**
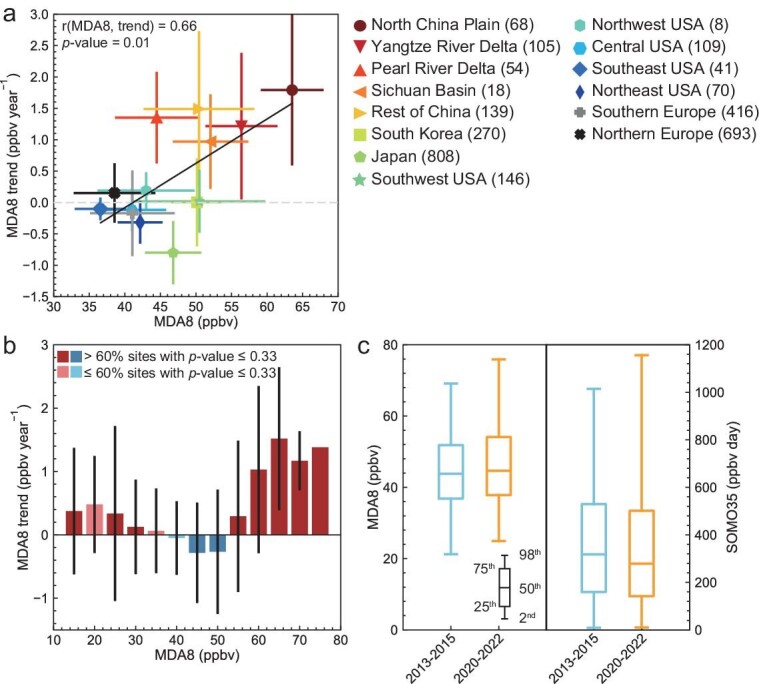
Mean MDA8 ozone level versus ozone trends during the warm seasons in 2013–2022. Trends are estimated using the generalized least-squares method based on monthly anomalies from 2013–2022 for all sites. Trends for each region are then calculated as the average of trends at all sites within the region. The horizontal and vertical bars indicate the standard deviation of MDA8 ozone trend and concentrations across all sites within the region for 2013–2022 in panel (a). The correlation coefficient between the regional site-averaged MDA8 ozone and trends is shown inset. Panel (b) shows the MDA8 ozone trends at different concentration bands (with a window of every 5 ppbv). The vertical bars indicate the standard deviation of the MDA8 ozone trend across all sites within the region for 2013–2022. Panel (c) shows the box whisker of MDA8 ozone and SOMO35, comparing 2013–2015 and 2020–2022. Fig. S5 shows the same comparison at the site level. Fig. S6 shows the same comparison in the cold season.

The spatial overlap of regions with both high levels of mean warm-season MDA8 ozone and increasing trends, as seen in Fig. 1, suggests that ozone pollution is continuously deteriorating over urban regions where ozone has already been at the highest level in the current monitoring network in the past decade. Figure 2 further illustrates this feature. We find a notable positive correlation (r = 0.66, *P*-value < 0.05) between the regional mean warm-season MDA8 ozone in 2013–2022 and ozone trends in the same period for the 14 regions across the northern hemisphere (Fig. 2a). The high correlation in the spatial distribution between the ozone levels and trends does not imply a direct causal relationship between the two, but it highlights the co-location of areas where ozone is high and increasing. We find that this feature mostly reflects the significant ozone increases over Chinese city clusters. The polluted urban regions with mean MDA8 ozone higher than 50 ppbv, including major city clusters in China, South Korea and SWUS, are all showing positive ozone trends. Ozone trends exceed 1.2 ppbv year^−1^ over the NCP and YRD city clusters where regional mean MDA8 ozone levels have been above 55 ppbv. Ozone increases in South Korea (<0.01 ppbv year^−1^) and SWUS (0.02 ± 0.5 ppbv year^−1^) are weaker, reflecting the variability in trends between individual sites, yet we have seen sites with high ozone levels showing large positive trends in these regions (e.g. the San Bernardino site with mean ozone of 70 ppbv and trends of 1.2 ppbv year^−1^). In comparison, less ozone-polluted urban regions with warm-season MDA8 ozone around or below 40 ppbv show negative ozone trends (SEUS, CUS, NEUS and Southern Europe) or weak ozone increases (NWUS and Northern Europe). Japan shows moderate ozone levels (47 ppbv in average) and the most prevailing negative trends. The difference in ozone trends in regions with high versus relatively low ozone levels suggests that ozone differences are growing between heavy-ozone-polluted and light-ozone-polluted urban regions.

This phenomenon is even more evident at the site level. We define polluted and clean sites as sites with MDA8 ozone levels at the upper and the lower fifth percentile of all 2945 sites, respectively. We find that mean MDA8 ozone trends at polluted sites (mean MDA8 ozone > 62 ppbv) are 1.2 ± 1.3 ppbv year^−1^, significantly larger than those for the clean sites (mean MDA8 ozone < 29 ppbv) of 0.2 ± 0.9 ppbv year^−1^. In particular, sites with MDA8 ozone >65 ppbv, including 29 sites in China and 3 sites in SWUS, all exhibit positive trends at a mean rate of 1.4 ppbv year^−1^ (Fig. 2b), effectively enlarging the ozone disparities between top-polluted sites and clean sites.

The growing ozone difference between heavy-ozone-polluted and light-ozone-polluted urban regions in 2013–2022 is not recorded by available observations in the other decades since 1980. Fig. S2 shows that before 2013, the NEUS and SWUS are the regions with the highest warm-season urban ozone levels on record. Ozone decreases in NEUS, while remaining relatively stable in SWUS since 1980. In comparison, ozone increases are mostly found in regions with relatively low ozone levels at that time, such as Japan and South Korea. These features shape a mostly constant or narrowing ozone difference between heavy-ozone-polluted and light-ozone-polluted urban regions before 2013. While national ozone monitoring in China is absent before 2013, the first-year ozone monitoring in China shows comparable ozone levels between the NCP and SWUS in 2013 (Fig. S2), suggesting that the ozone trends in China before 2013 should not affect the above ozone contrast. However, the rapid ozone increases in major Chinese city clusters with top ozone levels in 2013–2022 have significantly changed this pattern.

The larger ozone increases in heavy-ozone-polluted compared to light-ozone-polluted urban regions indicate that from the global perspective, the magnitude and frequency of extreme ozone pollution episodes have increased in the past decade. We find that the 98th percentile of warm-season daily urban MDA8 ozone levels increased from 69.1 ppbv in 2013–2015 to 75.9 ppbv in 2020–2022, with a 4-fold increase in the frequency of MDA8 ozone exceeding 80 ppbv. It also indicates an increasing difference in human and vegetation exposure to ozone between heavy-ozone-polluted and light-ozone-polluted regions, as reflected in a 13.9% expansion in the difference between the SOMO35 cumulative health exposure metric at the 98th versus that at the 2nd percentile from 2013–2015 to 2020–2022. More importantly, the regions with significantly increased human exposure to ozone also have high population densities, i.e. the NCP and YRD in China, indicating the growing threat of urban ozone to human health.

This phenomenon is not observed during the cold season (Fig. S5 and Fig. S6). We find no significant correlation between the mean MDA8 ozone level and trend in the regional mean (r = 0.2, n = 14, *P*-value = 0.49) and at site level (r = −0.02, n = 2945, *P*-value = 0.3), reflecting again a widespread increase in ozone in the cold season during 2013–2022 regardless of ozone pollution level.

### Revealing factors driving ozone increases from comparisons of decadal trends since 1980

**Figure 3.**
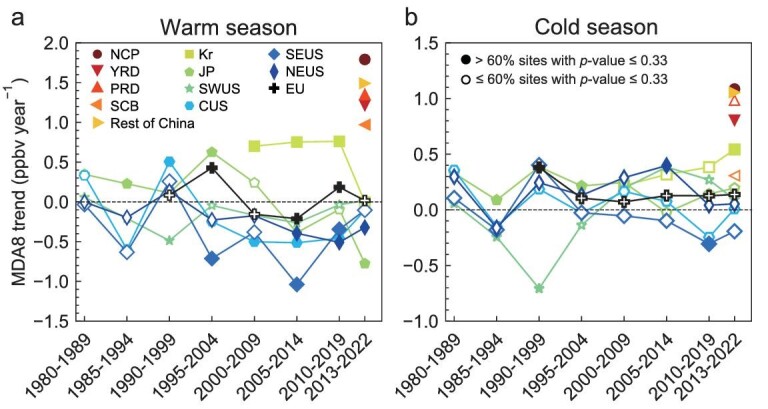
Mean MDA8 ozone trends in different decades and regions since 1980 in the northern hemisphere. (a and b) Decadal ozone trends based on monthly anomalies over 12 regions from several different time periods: (i) Europe for 1990–1999, 1995–2004, 2000–2009, 2005–2014, 2010–2019, 2013–2022; (ii) USA for 1980–1989, 1985–1994, 1990–1999, 1995–2004, 2000–2009, 2005–2014, 2010–2019, 2013–2022; (iii) South Korea for 2000–2009, 2005–2014, 2010–2019, 2013–2021; (iv) Japan for 1980–1989, 1985–1994, 1990–1999, 1995–2004, 2000–2009, 2005–2014, 2010–2019, 2013–2020; and (v) China for 2013–2022. Here we use sites with at least 60% of available monthly records in each decade to derive trends for MDA8 ozone during the warm and cold seasons. Since there are fewer observational sites in southern Europe, here, northern and southern Europe are combined into the whole of Europe. Fig. S8 shows the variation in the number of sites used in different regions. Fig. S7 shows the same trend variations but using continuous observation sites with at least 60% of available monthly records in 1980–2022.

**Figure 4.**
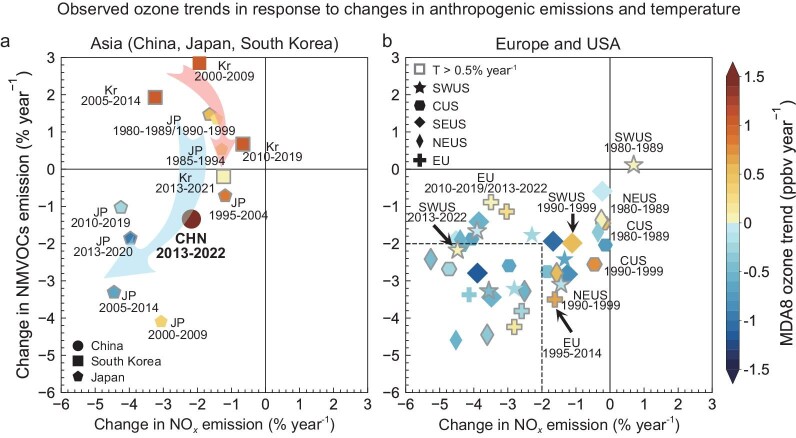
Links between historical ozone trends and contemporary trends in anthropogenic NO*_x_*, NMVOC emissions and temperature in different regions and periods since 1980. Here, only sites with *P*-values of decadal ozone trends ≤0.33 are used to provide robust relationships between ozone trends and anthropogenic emissions and temperature changes. Symbols with gray borders indicate that there is a significant positive temperature trend (>0.5% year^−1^) in this period. The record for 2013–2022 in China is highlighted. Table S4 displays the values shown in this figure.

**Figure 5.**
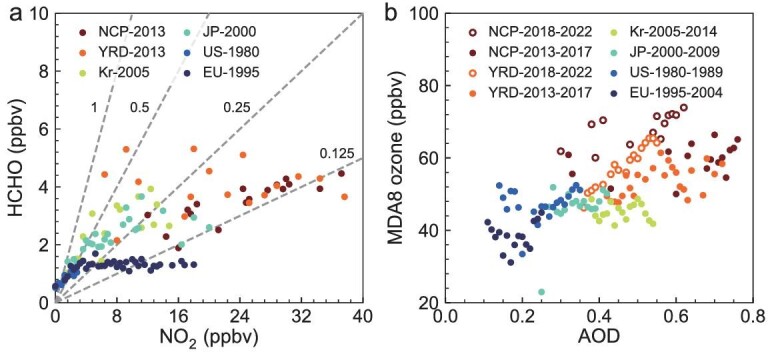
Distinct chemical environment of ozone formation in China during 2013–2022 compared to Japan, South Korea, Europe and the USA sites in their periods of high ozone concentration and/or increasing ozone pollution. Panel (a) shows the NO_2_ and HCHO levels for monitoring sites for each region. Data are obtained from a historical CESM2-WACCM simulation (Supplementary Data), re-sampled at the monitoring sites, and are binned and averaged over every 0.4 ppbv. The gray dashed lines represent the HCHO/NO_2_ ratio. Panel (b) shows the relationships between MDA8 ozone and AOD. AOD data are obtained from MERRA2 products (Materials and Methods), re-sampled at the monitoring sites, and are binned and averaged over an interval of 0.01.

The growing ozone difference in heavy-ozone-polluted versus light-ozone-polluted urban regions in 2013–2022, which is unprecedented in the observational network since 1980, largely reflects the faster ozone increase in city clusters in China than in other regions. Here, we compare the 2013–2022 ozone increases in China with decadal ozone trends in other regions and periods in history, alongside the concurrent trends in precursor emissions and temperature, to examine the similarity and disparity in the ozone trends and their driving factors from an observational perspective. This is crucial for understanding whether the rapid ozone increases in China in 2013–2022 have historical precedents and whether they are likely to continue in the future. Importantly, the historical evolution of ozone trends in different regions under anthropogenic emissions and climate change provides valuable references for future control strategies in regions experiencing current increases in ozone, such as China. We also conduct high-resolution, regional GEOS-Chem chemistry transport model calculations (horizontal resolution of 0.5°(latitude) × 0.625°(longitude)) to quantify the impact of emissions and weather conditions on ozone change in certain regions and periods (Materials and Methods). We first examine the variability of regional, decadal ozone trends since 1980 using different time windows and available ozone monitoring in Fig. 3. Decadal ozone trends at each site (site selections described in Materials and Methods) are calculated with a moving time window of five years and then averaged to represent regional mean trends. Figures 4 and 5 then link the decadal ozone trends to NO*_x_* and non-methane volatile organic compounds (NMVOCs) emission changes, surface temperature trends as an indicator of climate conditions, and aerosol concentration levels indicated by the aerosol optical depths (AOD). Data are summarized in Table S4.

Figure 3 shows large variability in decadal ozone trends since 1980 in each region, and that the 2013–2022 trends mostly do not follow those in previous decades. The decadal ozone trends in the central and eastern USA sites have been predominantly negative since 1985–1994, with trends intensifying in the 2000s, in agreement with previous studies [35,36]. The largest ozone decreases are recorded in 2005–2014 or 2010–2019, ranging from −1.0 to −0.34 ppbv year^−1^. These correspond to significant reductions in anthropogenic emissions of NO*_x_* and NMVOCs (Fig. S9) [37,38]. However, we find that the negative trends slow down in 2013–2022, even with the impact of COVID-19 included. This may reflect the influence of climate variability in the decadal ozone trend [39,40], as supported by the observed increase in warm-season temperature in 2013–2022. In the SWUS, the surface ozone decreases are weak compared to the eastern USA with a similar or even stronger reduction in NO*_x_* and NMVOCs emissions after the 1990s. The western USA has much higher background ozone than the eastern part [22,41,42]. The recent ozone increase in SWUS sites may also be tied to the positive temperature trends since 2005 [18]. In particular, the ozone-trend shift from negative to positive in 2013–2022 in SWUS may be partly attributed to the reported extreme high temperature and wildfires in 2019–2022 [43,44]. This is supported by our GEOS-Chem simulations that show meteorological conditions and biomass burning emissions during 2022 collectively increase surface ozone by 0.4 ± 1.1 ppbv across all US sites relative to emissions from 2013 (Fig. S13), consistent with recent studies [45,46]. In contrast, analyses of ozone concentrations at background or remote sites indicate an overall long-term increase in northern hemisphere baseline ozone levels since the 1980s, which stabilize or gradually decline after the mid-2000s [47,48]. Overall, ozone trends in urban sites do not align with the background or baseline ozone trends, suggesting that urban ozone trends are mostly modulated by local emission and chemistry. However, it is important to note that the potential effects of large-scale background ozone changes are difficult to separate completely from observed trends.

Warm-season ozone trends in Europe turn negative in the period of 2000–2009 and 2005–2014, in agreement with previous studies [49,50], but are positive during 2010–2019 and remain weakly positive in 2013–2022. Anthropogenic NO*_x_* and NMVOC emissions continue reducing during 2010–2019 but at a slower rate of 32.3% and 13.4%, respectively, compared to 39.0% and 29.3% in 2005–2014. We also find that Europe has experienced significant warming over the past four decades, with 2018 and 2022 ranking as two of the warmest years on record (Fig. S10). The regions with the highest temperatures and ozone anomalies in 2022 substantially overlap (Fig. S11). Europe also experienced extremely severe wildfires and drought in summer 2022. GEOS-Chem simulations show that wildfires in 2022 alone contributed to an ozone increase of 1.4 ± 1.8 ppbv at European sites compared to observed values in 2013. This suggests that changes in climate may complicate the link between decadal ozone trends and changes in anthropogenic emissions.

We see a plausible signal in the decadal ozone trend in Japan and South Korea in 2013–2022 compared to previous decades. In Japan, decadal ozone trends are predominately positive before 2000–2009, peaking at 0.62 ppbv year^−1^ in 1995–2004. Before 1999, Japan shows a lower surface HCHO to NO_2_ ratio (HCHO/NO_2_, see Materials and Methods) compared to the USA and Europe (Table S5), indicating a less NO*_x_*-sensitive environment for ozone formation. The positive ozone trends are associated with small decreases in NO*_x_* and increases in NMVOC emissions (Fig. S9). The positive ozone trend has since slowed down and become negative in and after 2005–2014 (-0.78 ppbv year^−1^ in 2013–2022) in response to joint decreases in NO*_x_* and NMVOC emissions (Fig. 3). Similarly, South Korea shows an ozone increase of 0.70–0.76 ppbv year^−1^ during 2000–2009, 2005–2014 and 2010–2019 when NO*_x_* emissions started to decrease but NMVOC emissions increased. The observed ozone increase is much weaker in 2013–2022, associated with a joint reduction in NO*_x_* and NMVOC emissions. GEOS-Chem simulations confirm the sensitivity of surface ozone over Japan and South Korea to reductions in anthropogenic emissions (Fig. S13). In Japan, a joint reduction in anthropogenic NO*_x_* and NMVOC emissions reduces surface ozone by 2.2 ± 0.7 ppbv over the period 2013–2022. In South Korea, simulated ozone increases by 0.8 ± 2.3 ppbv in 2005–2014, but the increase becomes much smaller at 0.1 ± 0.7 ppbv during 2013–2022 with reductions in both NO*_x_* and NMVOC emissions. Reductions in NMVOC emissions alone decrease ozone by 0.2 ± 0.08 ppbv over 2013–2022.

Figure 4 summarizes the response of the historical decadal ozone trends in the USA, Europe, Japan and South Korea to contemporary emission and temperature trends. We find that overall, the response of ozone trends to emissions and temperature changes is complex and non-linear; however, similarities in the response of ozone trends to emissions and temperature changes can still be summarized from these observations across different regions and time periods. First, the observed decadal ozone trends are overwhelmingly positive when anthropogenic NMVOC emissions increase, regardless of temperature trends. This quadrant covers decadal trends in Japan before 1995–2004 and in South Korea before 2010–2019. Increases in ozone are significantly reduced in South Korea, even turning negative in Japan, when NMVOC emissions begin to decrease, consistent with our model projections. This result highlights the importance of controlling NMVOC emissions in particular when NO*_x_* levels remain relatively high. Second, changes in surface temperature (and associated fire activity) largely modulates decadal ozone trends, in particular when reductions in anthropogenic emissions of ozone precursors are small (i.e. <2% year^−1^). Nevertheless, ozone trends are predominately negative when reductions in both anthropogenic NO*_x_* and NMVOC emissions are beyond 2% year^−1^, regardless of temperature trends (14 out of 17 cases), indicating that stringent controls of anthropogenic emissions are still effective to counteract ozone-favorable weather conditions. We find that temperature largely explains the variations in historical decadal ozone trends in the USA and Europe, where trends in anthropogenic emissions of NO*_x_* and NMVOCs have been predominately negative since the 1980s (Fig. 4).

We now place the 2013–2022 ozone trends in China in the context of worldwide historical records. Figure 3a shows that the observed warm-season ozone increases in Chinese city clusters of 1–1.8 ppbv year^−1^ during 2013–2022 are unprecedently high compared to decadal ozone trends in any other region with records, after 1980. Figure 4a shows that anthropogenic emissions of NO*_x_* and NMVOC emissions decrease by −2.2 and −1.3% year^−1^ during 2013–2022, and that temperature increases (0.5% year^−1^) in China are not significantly fast compared to other regions and periods. Overall, the comparison suggests that the ozone increase in China during 2013–2022 is unexpected, and seems to differ significantly in its magnitude and mechanisms from ozone increases observed in other countries and periods in history.

The ozone increases in China from 2013 to 2022 can be further divided into two phases: a rapid increase in ozone from 2013 to 2017 (18%) and a slower increase from 2018 to 2022 (2.3%). Since 2013, the Chinese government has implemented stringent emission reduction measures primarily targeting the control of fine particulate matter (PM_2.5_) pollution [51]. During the period of 2013–2017, anthropogenic emissions of NO*_x_* decreased by 20.9%, while anthropogenic emissions of NMVOCs increased by 2.9%. Ozone chemical productions in Chinese city clusters have been shown to be VOC-limited or mixed-sensitive due to high NO*_x_* levels [52,53] driven by intensive NO*_x_* emissions from fossil [54] and agricultural soil [55,56]. This is also reflected by the extremely high NO*_x_* and low HCHO/NO_2_ levels in the NCP region compared to Japan, South Korea, Europe and the US sites in periods of high ozone concentration and/or increasing ozone pollution (Fig. 5a). Therefore, the combination of decreasing NO*_x_* and increasing NMVOC emissions are expected to increase ozone concentration in the 2013–2017 period. This effect is not only supported by historical decadal ozone trends in other regions and periods (such as Japan in 1995–2004 and South Korea in 2000–2009 and 2005–2014), but also confirmed by studies relying on numerical simulations [11,54,57,58]. In the PRD region of China, where NO*_x_* emissions started to reduce earlier than NCP and YRD, studies have found that long-term trends (e.g. 2006–2021) of ozone are predominantly attributed to the decreased titration effect by NO [59,60].

As a consequence of the rapid decline in PM linked with reduced precursor emissions, surface ozone has increased rapidly in China due to a smaller loss of hydroperoxy radicals and increased solar radiation since 2013 [61,62]. Li *et al.* (2019) pointed out that this suppression effect is more pronounced at high PM concentrations [61]. Figure 5b compares the ozone-AOD relationships in the NCP and YRD city clusters of China from 2013 to 2022, with those in Japan, South Korea, Europe and the USA sites in their periods of high ozone concentration and/or increasing ozone pollution. We find AOD levels are high in the NCP and YRD regions, and that ozone and AOD levels typically showed a weak positive correlation, with more pronounced negative correlation when AOD exceeded 0.6 (i.e. regions with higher AOD levels show lower ozone concentration). A similar feature is also shown in South Korea during 2005–2014 but the overall AOD levels are much lower. In contrast, in the USA and Europe, even during periods of severe ozone pollution, AOD levels are typically lower than 0.4. In this regime, ozone and AOD levels show a notable positive correlation, reflecting their shared precursor sources and weather conditions. Under relatively low PM levels in Europe and the USA, decreases in PM levels with emission control do not lead to a significant increase in ozone. In contrast, China has significantly higher PM levels, as such decreases in PM levels with emission controls weaken the PM suppression effect on ozone and thus accelerate ozone formation. This represents a significant difference in ozone trends in China during the 2013–2022 period, especially the 2013–2017 period, compared to historical ozone trends in other countries and regions.

Since 2018, anthropogenic NMVOC emissions in China have started to decrease in response to the implementation of additional nationwide air pollution control measures. PM concentrations have further declined but remain relatively high as indicated by the AOD levels compared to historical records for other regions (Fig. 5b). Observations show that the positive trend in ozone has significantly weakened in 2018–2022 compared to 2013–2017 (Fig. S12), with the main increase occurring in 2019 and 2022. These two years had record-breakingly high summertime temperatures in China [14,63]. Our GEOS-Chem model simulations reveal that weather conditions in 2022 can explain an increase of 2.9∼4.5 ppbv in the YRD and PRD compared to ozone levels in 2013. Several studies have also pointed out that the more frequent ozone-favorable synoptic patterns during years 2019 and 2022 significantly enhance ozone concentrations and contribute to the overall increasing ozone trends in eastern China [64–66]. The above factors collectively contribute to the continued but fluctuating increase in ozone in 2018–2022. The weaker ozone increases in 2018–2022 compared to 2013–2017 also suggests that, with the progressively larger decrease in ozone precursor emissions and PM levels, we can anticipate that increases in ozone over China will become smaller and gradually reverse. This can be seen not only from the historical experience of Japan and South Korea, where the ozone trends gradually shifted from positive to negative after continuous reductions in anthropogenic NO*_x_* and NMVOC emissions, but also from the results of many model simulations predicting ozone decrease under emission reduction scenarios [57,67,68]. This is of significant importance in mitigating the future threat of ozone to human health and vegetation on a global scale.

In the cold season, the trends in decadal MDA8 ozone levels are dominantly positive since 1980s, except for the SEUS (Fig. 3b). This sustained upward trend has persisted through the years, with the period between 2013 and 2022 showing trends comparable or even surpassing those observed in earlier decades. Trends are particularly large in East Asia, where wintertime ozone formation tends to be more NO*_x_* saturated as indicated by the low HCHO/NO_2_ ratios, suggesting that current mitigations are not effective in terms of reducing the challenge posed to public health and crop production by the spread of ozone pollution into the boreal winter and spring seasons.

## SUMMARY AND DISCUSSION

In 2013–2022, there has been a rapid and extensive warm-season ozone increase in heavy-ozone-polluted urban regions, whereas trends are much weaker in light-ozone-polluted regions, effectively enlarging the ozone differences between the two regions. This is mostly driven by the fast ozone increases in Chinese city clusters that have had higher ozone levels and trends than any other region, since 1980. Ozone increases are also found at highly polluted sites in SWUS. The growing ozone difference reflects the increasing frequency and magnitude of extreme ozone pollution from a global perspective, and the increasing threat of ozone to human health and vegetation between the polluted and relatively clean urban areas.

We find that the magnitude of ozone increase over China during 2013–2022, and the underlying reasons for it, are different from other regions. These reasons are characterized by China's distinct chemical environment, with extremely high NO*_x_* levels (hence ozone formation is more NO*_x_* saturated) and high aerosol concentrations (which suppress ozone formation), in which reduction of precursor emissions, targeting NO*_x_* and PM, reversely increases ozone. However, new observations have shown that the ozone increase rate in China has slowed down in 2018–2022 compared to 2013–2017. Historical ozone evolution in other regions such as Japan and South Korea, where the ozone trends gradually shifted from positive to negative after continuous reductions in anthropogenic NO*_x_* and NMVOC emissions, suggests that ozone increase should ultimately be suppressed as emission reductions deepen. Our results also show the significant influence of weather anomalies (and associated extreme wildfires) on short-term ozone trends, especially when anthropogenic control of ozone precursors slows down, as evidenced by recent ozone trends in the USA and Europe. The rapid ozone increases in the cold seasons in all regions indicate a growing tendency for the aggregation of ozone pollution in winter and spring.

Our study thus constructs an intuitive framework to decipher the evolution of decadal urban ozone trends in different regions with contemporary emission and temperature changes. It also highlights the value of analyzing observed regional and temporal variability of historical decadal trends, in particular their response to changes in anthropogenic emissions and climate, for understanding the driving factors of ozone trends and paving the way for future ozone mitigation. Our findings call for further comprehensive and cross-national studies integrating field measurements and air quality models to probe into the underlying cause of ozone increases across different countries and periods.

## MATERIALS AND METHODS

### Data description

We collected hourly ozone observations from 1980 to 2022 (the most recent year of data currently available) from individual national monitoring networks, covering major developed and developing regions in the northern hemisphere, including China, the USA, Europe, South Korea, Japan and India (Fig. S1 and Table S1). We applied data quality control measures to exclude unreliable data (Supplementary Data) following our previous studies [69]. We used observations from urban and suburban sites to compare ozone levels and trends over different regions. We identified urban and suburban sites either by the national network or by information from the TOAR [70]. The monitoring network of the China National Environmental Monitoring Center is designed to monitor air pollution in urban and suburban areas. National monitoring networks in the USA, Europe, India, Japan and South Korea provided corresponding categories of stations based on their location. We calculated 10 ozone metrics for assessing ozone air quality and its impact on human health and the ecosystem following the TOAR definition (Table S2).

### Trends estimation

We derived the parametric linear trend of each ozone metric at each site for a specified 10-year period, using the generalized least-squares method with autocorrelation, following Cooper *et al.* [17] (Supplementary Data). We only used sites with at least 60% of available monthly records for each decade (i.e. >36 months in a decade of warm/cold seasons), and at least 4 valid monthly records in the first year of the decade, to derive trends for each ozone metric. For India, we calculated ozone metrics for individual stations for 2018–2021 since data for 2014–2017 did not meet the requirements of the previous trend calculations.

### GEOS-Chem chemical transport model

We conducted a total of 17 GEOS-Chem simulations (Table S6) to interpret the ozone change in certain periods, and to test the drivers of ozone difference by fixing the anthropogenic emissions, biomass burning emissions and/or meteorological conditions on a specific year. For example, to understand the drivers of ozone change between 2013 and 2022 in China, we first conducted simulations for 2013 (S1) and 2022 (S3) with anthropogenic emissions and meteorology at the respective year. We then conducted an additional simulation for the year 2013 but using an anthropogenic emissions inventory for China at the 2022 level (S2). The difference between S2 and S1 quantified the ozone difference due to emissions changes between years 2022 and 2013, and (S3 ‒ S1)-(S2 ‒ S1) quantified ozone change due to meteorology. We note that as the most recent emissions data extend only up to the year 2020, the ensuing sensitivity experiments substitute the 2022 emissions levels with those for 2020. Similar sensitivity simulations were conducted to quantify the impact of emissions changes on ozone in South Korea and Japan, and the impact of weather conditions and wildfires in Europe and the USA. The simulations were conducted for March–September for the specific years, and results for April–September were analyzed. Further details on the GEOS-Chem simulations are in Supplementary Data. Comparison with observed surface MDA8 ozone concentrations shows that GEOS-Chem simulation at a horizontal resolution of 0.5° × 0.625° well captures the spatial and temporal distributions of warm-season MDA8 ozone in China, Japan, South Korea, Europe and the USA, with high temporal correlation coefficients ranging from 0.63 to 0.84 between observed and simulated April–September daily MDA8 ozone in 2013 and 2022 across different regions. Nevertheless, the model shows moderate ozone high bias, ranging from 9 to 19 ppbv for China, 9 to 13 ppbv for Japan and South Korea, and 10 to 13 ppbv for the USA and Europe (Figs S14 and S15).

## Supplementary Material

nwae369_Supplemental_File

## Data Availability

The hourly surface observations of ozone over China, the USA, the European Union, South Korea, Japan and India are archived at https://quotsoft.net/air/, https://www.epa.gov/outdoor-air-quality-data, https://discomap.eea.europa.eu/map/fme/AirQualityExport.htm, https://airkorea.or.kr/web/, https://tenbou.nies.go.jp/download/ and https://app.cpcbccr.com/ccr/, respectively. The hourly 2 m air temperature MERRA-2 reanalysis data are from https://doi.org/10.5067/VJAFPLI1CSIV. The monthly mean AOD MERRA-2 reanalysis data are from https://doi.org/10.5067/XOGNBQEPLUC5. The updated global anthropogenic emissions data from the Community Emissions Data System (CEDS) are available from https://data.pnnl.gov/dataset/CEDS-4-21-21. The China Multi-Resolution Emission Inventory is available from http://meicmodel.org.cn/.

## References

[1] Unger N, Zheng Y, Yue X et al. Mitigation of ozone damage to the world's land ecosystems by source sector. Nat Clim Chang 2020; 10: 134–7. 10.1038/s41558-019-0678-3

[2] Bonell A, Sonko B, Badjie J et al. Environmental heat stress on maternal physiology and fetal blood flow in pregnant subsistence farmers in The Gambia, west Africa: an observational cohort study. Lancet Planet Health 2022; 6: e968–76. 10.1016/S2542-5196(22)00242-X 36495891 PMC9756110

[3] Feng Z, Xu Y, Kobayashi K et al. Ozone pollution threatens the production of major staple crops in East Asia. Nat Food 2022; 3: 47–56. 10.1038/s43016-021-00422-6 37118490

[4] Monks PS, Archibald AT, Colette A et al. Tropospheric ozone and its precursors from the urban to the global scale from air quality to short-lived climate forcer. Atmos Chem Phys 2015; 15: 8889–973. 10.5194/acp-15-8889-2015

[5] Fleming ZL, Doherty RM, von Schneidemesser E et al. Tropospheric Ozone Assessment Report: present-day ozone distribution and trends relevant to human health. Elem Sci Anth 2018; 6: 12. 10.1525/elementa.273

[6] Gaudel A, Cooper OR, Ancellet G et al. Tropospheric Ozone Assessment Report: present-day distribution and trends of tropospheric ozone relevant to climate and global atmospheric chemistry model evaluation. Elem Sci Anth 2018; 6: 39. 10.1525/elementa.291

[7] Zhang Y, Cooper OR, Gaudel A et al. Tropospheric ozone change from 1980 to 2010 dominated by equatorward redistribution of emissions. Nat Geosci 2016; 9: 875–9. 10.1038/ngeo2827 33117431 PMC7591124

[8] Wang H, Lu X, Jacob DJ et al. Global tropospheric ozone trends, attributions, and radiative impacts in 1995–2017: an integrated analysis using aircraft (IAGOS) observations, ozonesonde, and multi-decadal chemical model simulations. Atmos Chem Phys 2022; 22: 13753–82. 10.5194/acp-22-13753-2022

[9] Wang W, Parrish DD, Wang S et al. Long-term trend of ozone pollution in China during 2014–2020: distinct seasonal and spatial characteristics and ozone sensitivity. Atmos Chem Phys 2022; 22: 8935–49. 10.5194/acp-22-8935-2022

[10] Wang T, Xue L, Feng Z et al. Ground-level ozone pollution in China: a synthesis of recent findings on influencing factors and impacts. Environ Res Lett 2022; 17: 063003. 10.1088/1748-9326/ac69fe

[11] Liu Y, Geng G, Cheng J et al. Drivers of increasing ozone during the two phases of Clean air Actions in China 2013–2020. Environ Sci Technol 2023; 57: 8954–64. 10.1021/acs.est.3c00054 37276527 PMC10286302

[12] Zheng B, Tong D, Li M et al. Trends in China's anthropogenic emissions since 2010 as the consequence of clean air actions. Atmos Chem Phys 2018; 18: 14095–111. 10.5194/acp-18-14095-2018

[13] Lu X, Zhang L, Wang X et al. Rapid increases in warm-season surface ozone and resulting health impact in China since 2013. Environ Sci Technol Lett 2020; 7: 240–7. 10.1021/acs.estlett.0c00171

[14] Li K, Jacob DJ, Shen L et al. Increases in surface ozone pollution in China from 2013 to 2019: anthropogenic and meteorological influences. Atmos Chem Phys 2020; 20: 11423–33. 10.5194/acp-20-11423-2020

[15] Parrish DD, Derwent RG, Faloona IC et al. Technical note: northern midlatitude baseline ozone—long-term changes and the COVID-19 impact. Atmos Chem Phys 2022; 22: 13423–30. 10.5194/acp-22-13423-2022

[16] Han H, Zhang L, Liu Z et al. Narrowing differences in urban and nonurban surface ozone in the Northern Hemisphere over 1990–2020. Environ Sci Technol Lett 2023; 10: 410–17. 10.1021/acs.estlett.3c00105

[17] Cooper OR, Schultz MG, Schröder S et al. Multi-decadal surface ozone trends at globally distributed remote locations. Elem Sci Anth 2020; 8: 23. 10.1525/elementa.420

[18] Lin M, Horowitz LW, Payton R et al. US surface ozone trends and extremes from 1980 to 2014: quantifying the roles of rising Asian emissions, domestic controls, wildfires, and climate. Atmos Chem Phys 2017; 17: 2943–70. 10.5194/acp-17-2943-2017

[19] Lu X, Hong J, Zhang L et al. Severe surface ozone pollution in China: a global perspective. Environ Sci Technol Lett 2018; 5: 487–94. 10.1021/acs.estlett.8b00366

[20] Koplitz S, Simon H, Henderson B et al. Changes in ozone chemical sensitivity in the United States from 2007 to 2016. ACS Environ Au 2022; 2: 206–22. 10.1021/acsenvironau.1c00029 35967933 PMC9371464

[21] Wang N, Xu J, Pei C et al. Air quality during COVID-19 lockdown in the Yangtze River Delta and the Pearl River Delta: two different responsive mechanisms to emission reductions in China. Environ Sci Technol 2021; 55: 5721–30. 10.1021/acs.est.0c08383 33797897

[22] Jaffe DA, Cooper OR, Fiore AM et al. Scientific assessment of background ozone over the U.S.: implications for air quality management. Elem Sci Anth 2018; 6: 56. 10.1525/elementa.309 PMC6198683 30364819

[23] Lu X, Zhang L, Yue X et al. Wildfire influences on the variability and trend of summer surface ozone in the mountainous western United States. Atmos Chem Phys 2016; 16: 14687–702. 10.5194/acp-16-14687-2016

[24] Lu X, Zhang L, Liu X et al. Lower tropospheric ozone over India and its linkage to the South Asian monsoon. Atmos Chem Phys 2018; 18: 3101–18. 10.5194/acp-18-3101-2018

[25] Gao M, Gao J, Zhu B et al. Ozone pollution over China and India: seasonality and sources. Atmos Chem Phys 2020; 20: 4399–414. 10.5194/acp-20-4399-2020

[26] Yin H, Lu X, Sun Y et al. Unprecedented decline in summertime surface ozone over eastern China in 2020 comparably attributable to anthropogenic emission reductions and meteorology. Environ Res Lett 2021; 16: 124069. 10.1088/1748-9326/ac3e22

[27] Wakamatsu S, Morikawa T, Ito A. Air pollution trends in Japan between 1970 and 2012 and impact of urban air pollution countermeasures. Asian J Atmos Environ 2013; 7: 177–90. 10.5572/ajae.2013.7.4.177

[28] Akimoto H, Mori Y, Sasaki K et al. Analysis of monitoring data of ground-level ozone in Japan for long-term trend during 1990–2010: causes of temporal and spatial variation. Atmos Environ 2015; 102: 302–10. 10.1016/j.atmosenv.2014.12.001

[29] Le T, Wang Y, Liu L et al. Unexpected air pollution with marked emission reductions during the COVID-19 outbreak in China. Science 2020; 369: 702–6. 10.1126/science.abb7431 32554754 PMC7402623

[30] Li K, Jacob DJ, Liao H et al. Ozone pollution in the North China Plain spreading into the late-winter haze season. Proc Natl Acad Sci USA 2021; 118: e2015797118. 10.1073/pnas.2015797118 33649215 PMC7958175

[31] Doumbia T, Granier C, Elguindi N et al. Changes in global air pollutant emissions during the COVID-19 pandemic: a dataset for atmospheric modeling. Earth Syst Sci Data 2021; 13: 4191–206. 10.5194/essd-13-4191-2021

[32] Cooper MJ, Martin RV, Hammer MS et al. Global fine-scale changes in ambient NO_2_ during COVID-19 lockdowns. Nature 2022; 601: 380–7. 10.1038/s41586-021-04229-0 35046607 PMC8770130

[33] Keller CA, Evans MJ, Knowland KE et al. Global impact of COVID-19 restrictions on the surface concentrations of nitrogen dioxide and ozone. Atmos Chem Phys 2021; 21: 3555–92. 10.5194/acp-21-3555-2021

[34] Lee H-M, Park RJ. Factors determining the seasonal variation of ozone air quality in South Korea: regional background versus domestic emission contributions. Environ Pollut 2022; 308: 119645. 10.1016/j.envpol.2022.119645 35718046

[35] Simon H, Reff A, Wells B et al. Ozone trends across the United States over a period of decreasing NOx and VOC emissions. Environ Sci Technol 2015; 49: 186–95. 10.1021/es504514z 25517137

[36] Strode SA, Rodriguez JM, Logan JA et al. Trends and variability in surface ozone over the United States. JGR Atmos 2015; 120: 9020–42. 10.1002/2014JD022784

[37] Li P, Yang Y, Wang H et al. Source attribution of near-surface ozone trends in the United States during 1995–2019. Atmos Chem Phys 2023; 23: 5403–17. 10.5194/acp-23-5403-2023

[38] Xing J, Pleim J, Mathur R et al. Historical gaseous and primary aerosol emissions in the United States from 1990 to 2010. Atmos Chem Phys 2013; 13: 7531–49. 10.5194/acp-13-7531-2013

[39] Fiore AM, Milly GP, Hancock SE et al. Characterizing changes in Eastern U.S. pollution events in a warming world. JGR Atmos 2022; 127: e2021JD035985. 10.1029/2021JD035985

[40] Lin M, Fiore AM, Horowitz LW et al. Climate variability modulates western US ozone air quality in spring via deep stratospheric intrusions. Nat Commun 2015; 6: 7105. 10.1038/ncomms8105 25964012 PMC4432627

[41] Cooper OR, Langford AO, Parrish DD et al. Challenges of a lowered U.S. ozone standard. Science 2015; 348: 1096–7. 10.1126/science.aaa5748 26045425

[42] Zhang L, Jacob DJ, Yue X et al. Sources contributing to background surface ozone in the US Intermountain West. Atmos Chem Phys 2014; 14: 5295–309. 10.5194/acp-14-5295-2014

[43] Pan K, Faloona IC. The impacts of wildfires on ozone production and boundary layer dynamics in California's Central Valley. Atmos Chem Phys 2022; 22: 9681–702. 10.5194/acp-22-9681-2022

[44] Iglesias V, Balch JK, Travis WR. U.S. fires became larger, more frequent, and more widespread in the 2000s. Sci Adv 2022; 8: eabc0020. 10.1126/sciadv.abc0020 35294238 PMC8926334

[45] Peischl J, Aikin KC, McDonald BC et al. Quantifying anomalies of air pollutants in 9 U.S. cities during 2020 due to COVID-19 lockdowns and wildfires based on decadal trends. Elem Sci Anth 2023; 11: 00029. 10.1525/elementa.2023.00029

[46] Putero D, Cristofanelli P, Chang KL et al. Fingerprints of the COVID-19 economic downturn and recovery on ozone anomalies at high-elevation sites in North America and western Europe. Atmos Chem Phys 2023; 23: 15693–709. 10.5194/acp-23-15693-2023 PMC12181938 40548228

[47] Parrish DD, Derwent RG, Faloona IC. Long-term baseline ozone changes in the Western US: a synthesis of analyses. J Air Waste Manage Assoc 2021; 71: 1397–406. 10.1080/10962247.2021.1945706 34166173

[48] Parrish DD, Derwent RG, Staehelin J. Long-term changes in northern mid-latitude tropospheric ozone concentrations: synthesis of two recent analyses. Atmos Environ 2021; 248: 118227. 10.1016/j.atmosenv.2021.118227

[49] Lin M, Horowitz LW, Xie Y et al. Vegetation feedbacks during drought exacerbate ozone air pollution extremes in Europe. Nat Clim Chang 2020; 10: 444–51. 10.1038/s41558-020-0743-y

[50] Yan Y, Pozzer A, Ojha N et al. Analysis of European ozone trends in the period 1995–2014. Atmos Chem Phys 2018; 18: 5589–605. 10.5194/acp-18-5589-2018

[51] Zhai S, Jacob DJ, Wang X et al. Fine particulate matter (PM_2.5_) trends in China, 2013–2018: separating contributions from anthropogenic emissions and meteorology. Atmos Chem Phys 2019; 19: 11031–41. 10.5194/acp-19-11031-2019

[52] Jin X, Holloway T. Spatial and temporal variability of ozone sensitivity over China observed from the Ozone Monitoring Instrument. JGR Atmos 2015; 120: 7229–46. 10.1002/2015JD023250

[53] Wang W, van der A R, Ding J et al. Spatial and temporal changes of the ozone sensitivity in China based on satellite and ground-based observations. Atmos Chem Phys 2021; 21: 7253–69. 10.5194/acp-21-7253-2021

[54] Wang Y, Zhao Y, Liu Y et al. Sustained emission reductions have restrained the ozone pollution over China. Nat Geosci 2023; 16: 967–74. 10.1038/s41561-023-01284-2

[55] Tan W, Wang H, Su J et al. Soil emissions of reactive nitrogen accelerate summertime surface ozone increases in the North China Plain. Environ Sci Technol 2023; 57: 12782–93. 10.1021/acs.est.3c01823 37596963

[56] Lu X, Ye X, Zhou M et al. The underappreciated role of agricultural soil nitrogen oxide emissions in ozone pollution regulation in North China. Nat Commun 2021; 12: 5021. 10.1038/s41467-021-25147-9 34408153 PMC8373933

[57] Li K, Jacob DJ, Liao H et al. Anthropogenic drivers of 2013–2017 trends in summer surface ozone in China. Proc Natl Acad Sci USA 2019; 116: 422–7. 10.1073/pnas.1812168116 30598435 PMC6329973

[58] Lyu X, Li K, Guo H et al. A synergistic ozone-climate control to address emerging ozone pollution challenges. One Earth 2023; 6: 964–77. 10.1016/j.oneear.2023.07.004

[59] Zeng L, Yang J, Guo H et al. Impact of NO_x_ reduction on long-term surface ozone pollution in roadside and suburban Hong Kong: field measurements and model simulations. Chemosphere 2022; 302: 134816. 10.1016/j.chemosphere.2022.134816 35525456

[60] Li X-B, Yuan B, Parrish DD et al. Long-term trend of ozone in southern China reveals future mitigation strategy for air pollution. Atmos Environ 2022; 269: 118869. 10.1016/j.atmosenv.2021.118869

[61] Li K, Jacob DJ, Liao H et al. A two-pollutant strategy for improving ozone and particulate air quality in China. Nat Geosci 2019; 12: 906–10. 10.1038/s41561-019-0464-x

[62] Ivatt PD, Evans MJ, Lewis AC. Suppression of surface ozone by an aerosol-inhibited photochemical ozone regime. Nat Geosci 2022; 15: 536–40. 10.1038/s41561-022-00972-9

[63] Zhang T, Deng Y, Chen J et al. An energetics tale of the 2022 mega-heatwave over central-eastern China. npj Clim Atmos Sci 2023; 6: 162. 10.1038/s41612-023-00490-4

[64] Liu N, He G, Wang H et al. Rising frequency of ozone-favorable synoptic weather patterns contributes to 2015–2022 ozone increase in Guangzhou. J Environ Sci 2025; 148: 502–14. 10.1016/j.jes.2023.09.024 39095184

[65] Hu T, Lin Y, Liu R et al. What caused large ozone variabilities in three megacity clusters in eastern China during 2015–2020? Atmos Chem Phys 2024; 24: 1607–26. 10.5194/acp-24-1607-2024

[66] Hu W, Liu R, Chen Z et al. Processes conducive to high ozone formation in Pearl River Delta in the presence of Pacific tropical cyclones. Atmos Environ 2023; 307: 119859. 10.1016/j.atmosenv.2023.119859

[67] Liu Y, Wang T. Worsening urban ozone pollution in China from 2013 to 2017–Part 2: the effects of emission changes and implications for multi-pollutant control. Atmos Chem Phys 2020; 20: 6323–37. 10.5194/acp-20-6323-2020

[68] Wang W, Li X, Cheng Y et al. Ozone pollution mitigation strategy informed by long-term trends of atmospheric oxidation capacity. Nat Geosci 2024; 17: 20–5. 10.1038/s41561-023-01334-9

[69] Wang H, Wang H, Lu X et al. Increased night-time oxidation over China despite widespread decrease across the globe. Nat Geosci 2023; 16: 217–23. 10.1038/s41561-022-01122-x

[70] Schultz MG, Schröder S, Lyapina O et al. Tropospheric Ozone Assessment Report: database and metrics data of global surface ozone observations. Elem Sci Anth 2017; 5: 58. 10.1525/elementa.244

