# Supplementary material for: Deciphering decadal urban ozone trends from historical records since 1980
Source: Natl Sci Rev. 2024 Oct 24;11(11):nwae369. doi: 10.1093/nsr/nwae369 (PMC11585278; doi:10.1093/nsr/nwae369)
Supplement: nwae369_Supplemental_File [file nwae369_supplemental_file.docx]

Supporting Information for

**Deciphering decadal urban ozone trends from historical records since 1980**

Haolin Wang, Xiao Lu^*^, Paul I. Palmer, Lin Zhang, Keding Lu, Ke Li, Tatsuya Nagashima, Ja-Ho Koo, Hiroshi Tanimoto, Haichao Wang, Meng Gao, Cheng He, Kai Wu, Shaojia Fan^*^, Yuanhang Zhang^*^

*To whom correspondence should be addressed.

Email: [luxiao25@mail.sysu.edu.cn](mailto:luxiao25@mail.sysu.edu.cn) (X. L.), [eesfsj@mail.sysu.edu.cn](mailto:eesfsj@mail.sysu.edu.cn) (S. F.), [yhzhang@pku.edu.cn](mailto:yhzhang@pku.edu.cn) (Y. Z.)

**This PDF file includes:**

Supporting Text S1

Supporting Figures. S1 to S15

Supporting Tables. S1 to S6

Text S1

**1.1 Data quality control for the hourly data**

We performed data quality control on the hourly surface ozone data by applying guidelines to exclude unreliable data outliers. The continuously observed stations were selected for trend analysis, and the ozone measurements were converted to a mixing ratio at local time (ppbv). Our quality control guidelines were mainly based on Wang et al. [1] and included five criteria for excluding hourly outliers:

(1) ozone values greater than 500 ppbv or less than 0 ppbv,

(2) hourly ozone normalized values greater than 5,

(3) daily variability less than 2 ppbv (the difference between the daily maximum and minimum values is less than 2 ppbv),

(4) the same value for at least 4 out of 5 consecutive hours,

(5) unrealistically huge spikes.

After applying the quality control measures, 3.7%, 6.7%, 6.3%, 5.3%, 6.9%, and 9.5% of hourly records were removed for China, USA, EU, Japan, South Korea, and India, respectively, for the period from 2013 to the most recent available observations.

**1.2 Ozone Metrics**

We calculate ten ozone metrics for assessing ozone air quality and its impact on human health and the ecosystem following the TOAR definition [2] (Table S2). The metrics include the 5th (Perc5), 50th (Perc50), and 95th-percentile (Perc95) ozone levels, daytime and nighttime averages, mean daily 8-h average maximum (MDA8), and the total number of days with MDA8 values>70 ppbv (NDGT70), and acculative metrics used to assess human health and ecosystem exposure impacts. MDA8 is the most widely used daily ozone metric for air quality regulation in many countries. Health exposures are assessed through the short-term human exposure metric (e.g., MDA8 and NDGT70), or through metrics that consider cumulative exposure (e.g., SOMO35, the sum of daily MDA8 ozone over 35 ppbv). AOT40 and W126 metrics are used to estimate ozone damage to vegetation. We calculate all metrics from hourly measurements, following quality control based on TOAR data completeness requirements and procedures (Table S2).

**1.3 Trends estimation**

For environmental time series, such as ozone, that have a strong seasonal cycle, autocorrelation, and perhaps periods of missing data [3], estimated trends determined from monthly mean anomalies are more accurate than using the monthly mean data. We first derive the monthly mean anomalies of each ozone metric by subtracting the monthly decadal-mean values from the monthly mean data. We then estimate linear trends using the following generalized least squares method with autoregression [1, 4]:

$y_{t}=b+kt+\alpha\cos\left( {2\pi M}/6 \right)+\beta\sin\left( {2\pi M}/6 \right)+AR_{t}$ (1)

where $y_{t}$ represents the ozone metrics monthly anomaly in month $t$, which is the index of the month during the decade (i.e., from 1 to 60), $b$ indicates the intercept, $k$ is the linear trend coefficient, $\alpha$ and $\beta$ are coefficients for a 6-month harmonic series of seasonal cycle (M ranges from 1 to 6) and $AR_{t}=\rho{AR}_{t-1}+ \epsilon_{t}$ accounts for autocorrelation with $\epsilon_{t}$ representing a normal random error series. Trend reliability can be expressed with the p-value. A p-value > 0.33 is used to indicate a trend with very low certainty or no evidence, which has been applied in TOAR Phase II [5]. We also use the Theil−Sen estimator to calculate the MDA8 ozone trends and Mann-Kendall tests to derive the p-values [6, 7], which has been applied in TOAR and other ozone trend studies. The parametric linear trend estimates are consistent with the Theil−Sen trend estimates (Table S3), and we report parametric linear trend estimates in the main text.

**1.4 Trends estimation for anthropogenic NO*_x_* and NMVOCs emissions, temperature, and aerosol optical depth.**

In this study we used the global anthropogenic emissions inventory from the Community Emissions Data System (CEDS), which produces consistent estimates of global air emissions species over the industrial era (1750 - present). We used the most recent version of CEDS emissions including gridded emissions data for NO*_x_* and NMVOCs from 1980-2020 mapped to a 0.5° global grid. In China, we utilize the Multi-resolution Emission Inventory for China (MEIC) anthropogenic emissions inventory instead of CEDS for the period spanning 1990 to 2020. The hourly air temperature at a 2 m above ground level and aerosol optical depth (AOD) were acquired from the National Aeronautics and Space Administration's (NASA) Modern-Era Retrospective Analysis for Research and Applications, version 2 (MERRA-2) product, with a spatial resolution of 0.5° × 0.625°. We resample the gridded data in accordance with the geographical locations of the ozone monitoring sites across different regions, ensuring congruence with the observed ozone data, and then averaged across distinct time windows and regions. Their trends in Fig. 3 and Fig. 4 are computed in the same way as the trends in the ozone metrics. The trend in percentage is then obtained by dividing the linear trend by the average in the first year.

**1.5 Surface HCHO to NO_2_ ratio (****HCHO/NO_2_)**

The ratio of HCHO to NO_2_ serves as an indicator ozone chemical regime and its sensitivity to NO*_x_* and NMVOCs emissions [8]. This ratio is computed through the resampling of historical CESM2-WACCM simulation (1850-2014) at a resolution of 0.9°×1.25° to observation sites across diverse regions. The model outputs are available on the Earth System Grid Federation (ESGF). A higher ratio of HCHO to NO_2_ suggests heightened sensitivity of ozone formation to variations in NO*_x_* emissions compared to NMVOCs emissions.

**1.6 Sites selection for long-term trend analyses.**

Fig. 3 and Fig. 4 utilize ozone observations from 1980. The number of sites with available observations to derive decadal trends is not consistent throughout decades in 1980–2022 (Fig. S8). We employ two methodologies to calculate ozone trends at each site. The first only includes sites with continuous observations during 1980–2022, i.e., with a sufficient data length of measurement to derive trends in all decades during 1980–2022. This significantly reduces the number of sites for analyses. The second utilizes all available sites with a sufficient length of measurement for each designated time window, so the number of sites differs between each decade. We find that both methods yield consistent trends for all regions except for the SWUS region (second method in Fig. 3 vs first method in Fig. S7). We adopt the second method to allow more sites to be included in the analyses.

**1.7 GEOS-Chem Chemical Transport Model.**

We employ the global three-dimensional chemical transport model GEOS-Chem version 13.3.1 (available at https://github.com/geoschem/GCClassic/tree/13.3.1, last assessed 20 July 2023) [9] to interpret warm-season ozone trends in East Asia, Europe, and the US and to explore the role of natural factors (meteorology and biomass burning) and emissions. The model is driven by Goddard Earth Observing System, version 5 (GEOS-5) Modern-Era Retrospective analysis for Research and Application version 2 (MERRA-2) assimilated meteorological fields. We perform the nested-grid simulation at a high spatial resolution over China, South Korea, Europe, and the US at 0.5°(latitude)×0.625°(longitude) resolution, with boundary conditions archived from consistent global simulations at 4°×5° resolution [10]. Our model configuration largely follows Wang et al. (2022), but we use a higher spatial resolution and apply anthropogenic emissions from the latest version of the Community Emission Data System inventory (CEDSv-2021-04-21). In China, we utilize the Multi-resolution Emission Inventory for China (MEIC) anthropogenic emissions inventory instead of CEDS for the period spanning 1990 to 2020[11].

Table S1. Information of hourly ozone observations collected by individual national monitoring networks.

| Country | Number of urban and suburban sites | Measurement period | Source agent | Source URLs |
| --- | --- | --- | --- | --- |
| China | 2028 | 2013–2022 | China National Environmental Monitoring Center | http://106.37.208.233:20035/ |
| United States | 1681 | 1980–2022 | Environmental Protection Agency Air Quality System monitoring network | https://aqs.epa.gov/aqsweb/airdata/download_files.html |
| Europe | 2240 | 2013–2022 | European Environment Agency | https://discomap.eea.europa.eu/map/fme/AirQualityExport.htm |
|  | 1915 | 1990–2012 | European Environment Agency | https://www.eea.europa.eu/data-and-maps/data/airbase-the-european-air-quality-database-8 |
| South Korea | 554 | 2001–2022 | Korean Ministry of Environment | https://airkorea.or.kr/ |
| Japan | 899 | 1980–2020 | National Institute for Environmental Studies | https://tenbou.nies.go.jp/download/ |
| India | 267 | 2014–2021 | Central Control Room for Air Quality Management | https://app.cpcbccr.com/ccr/ |

Table S2. Description of ozone metrics.

| Metrics | Definition | Calculation methods |
| --- | --- | --- |
| MDA8  (ppbv) | daily maximum 8 h average | There should be 75% valid hourly records to calculate the MDA8. |
| DTAvg  (ppbv) | average of hourly ozone concentrations for the 12 hours from 08:00 to 19:59 local time | There should be 75% valid hourly records to calculate the daily DTAvg. |
| NTAvg  (ppbv) | average of hourly ozone concentrations for the 12 hours from 20:00 to 07:59 the next day local time | There should be 75% valid hourly records to calculate the daily NTAvg. |
| Perc5  (ppbv) | 5^th^ percentile of hourly concentrations | There should be 75% valid hourly records to calculate the daily Perc5. |
| Perc50  (ppbv) | 50^th^ percentile of hourly concentrations | There should be 75% valid hourly records to calculate the daily Perc50. |
| Perc95  (ppbv) | 95^th^ percentile of hourly concentrations | There should be 75% valid hourly records to calculate the daily Perc95. |
| NDGT70  (day) | total number of days with MDA8 values of >70 ppbv | There should be 75% valid daily records to calculate the daily NDGT70. |
| AOT40  (ppbv h) | cumulative daytime hourly ozone concentrations of >40 ppbv | There should be 75% valid daily records. Daily AOT40 is scaled by the fraction (Ntotal/Nvalid), where Ntotal denotes 12 hours and Nvalid denotes the number of valid records. |
| SOMO35  (ppbv day) | the sum of positive differences between the daily MDA8 and the cut-off concentration set at 35 ppbv | At least 75% valid daily records are required to calculate the monthly SOMO35. Furthermore, the monthly SOMO35 will be scaled by the fraction (Ntotal/Nvalid), where Ntotal denotes the number of the month and Nvalid denotes the number of valid records. |
| W126  (ppbv h) | daily W126 is calculated using the formula W126 = $\sum_{i} w_{i}C_{i}$, where $C_{i}$ denotes the hourly ozone concentration in ppbv for the 12 h period from 08:00 to 19:59 local time and $w_{i}$ is the weighting index defined as $w_{i}=\frac{1}{1+M\times exp(-AC_{i}/1000)}$, where M = 4403 and A = 126 | The same data criteria as AOT40 is applied. |

Table S3. 2013–2022 MDA8 ozone trends averaged over 13 regions across the Northern Hemisphere.

|  | Regions | Warm season | | Cold season | |
| --- | --- | --- | --- | --- | --- |
|  |  | MDA8 trend  [ppbv year^-1^] | Number of positive sites vs negative sites | MDA8 trend  [ppbv year^-1^] | Number of positive sites vs negative sites |
| Linear trend | NCP | 1.8 | 64 (59) : 4 (2) | 1.1 | 66 (58) : 2 (0) ^a^ |
|  | YRD | 1.2 | 98 (86) : 7 (3) | 0.8 | 99 (84) : 6 (2) |
|  | PRD | 1.4 | 54 (48) : 0 (0) | 1.0 | 48 (33) : 6 (2) |
|  | SCB | 1.0 | 17 (16) : 1 (1) | 0.3 | 15 (7) : 3 (0) |
|  | Kr | 0.003 | 136 (78) : 134 (68) | 0.6 | 233 (179) : 37 (12) |
|  | JP | -0.8 | 49 (12) : 759 (615) | 0.2 | 586 (221) : 222 (36) |
|  | SEU | -0.2 | 141 (86) : 275 (197) | 0.03 | 238 (96) : 178 (71) |
|  | NEU | 0.2 | 488 (259) : 205 (94) | 0.2 | 563 (258) : 130 (38) |
|  | SWUS | 0.02 | 79 (41) : 67 (36) | 0.1 | 101 (41) : 45 (12) |
|  | NWUS | 0.2 | 7 (3) : 1 (1) | 0.3 | 7 (4) : 1 (1) |
|  | CUS | -0.1 | 35 (16) : 74 (39) | -0.01 | 48 (23) : 61 (21) |
|  | SEUS | -0.1 | 12 (2) : 29 (3) | -0.2 | 7 (1) : 34 (12) |
|  | NEUS | -0.3 | 9 (5) : 61 (47) | 0.05 | 38 (13) : 32 (9) |
| Theil-Sen trend | NCP | 1.7 | 64 (55) : 4 (2) | 1.1 | 66 (61) : 2 (0) |
|  | YRD | 1.2 | 101 (84) : 4 (3) | 0.9 | 98 (89) : 7 (2) |
|  | PRD | 1.2 | 53 (45) : 1 (0) | 0.9 | 47 (35) : 7 (2) |
|  | SCB | 1.1 | 17 (17) : 1 (1) | 0.3 | 14 (9) : 4 (0) |
|  | Kr | -0.01 | 130 (76) : 140 (72) | 0.6 | 235 (185) : 35 (12) |
|  | JP | -0.9 | 47 (9) : 761 (624) | 0.2 | 611 (244) : 197 (37) |
|  | SEU | -0.2 | 143 (77) : 273 (191) | 0.02 | 237 (88) : 179 (73) |
|  | NEU | 0.1 | 470 (254) : 223 (103) | 0.2 | 537 (225) : 156 (44) |
|  | SWUS | 0.02 | 75 (40) : 71 (36) | 0.1 | 98 (35) : 48 (9) |
|  | NWUS | 0.1 | 6 (2) : 2 (1) | 0.3 | 7 (4) : 1 (1) |
|  | CUS | -0.1 | 35 (14) : 74 (41) | 0.01 | 52 (21) : 57 (17) |
|  | SEUS | -0.2 | 7 (1) : 34 (8) | -0.1 | 10 (2) : 31 (10) |
|  | NEUS | -0.3 | 10 (5) : 60 (45) | 0.03 | 36 (12) : 34 (8) |

^a^ Values in the parentheses are numbers of sites with *p*-values<=0.33.

**Table S4.** Links of historical ozone trends to contemporary trends in anthropogenic NO*_x_*, NMVOCs emissions, temperature, and AOD in different regions and periods since 1980.

| Region | Year | MDA8  [ppbv/year] | NO*_x_*  [%/year] | NMVOCs  [%/year] | T ^a^  [%/year] | AOD  [%/year] |
| --- | --- | --- | --- | --- | --- | --- |
| CHN | 2013-2022 | 1.60 | -2.17 | -1.34 | 0.47 | -3.16 |
| Kr | 2000-2009 | 1.05 | -1.94 | 2.85 | 0.47 | 1.76 |
|  | 2005-2014 | 1.02 | -3.24 | 1.93 | 0.73 | -1.42 |
|  | 2010-2019 | 1.05 | -0.66 | 0.67 | 1.35 | -3.87 |
|  | 2013-2022 | 0.02 | -1.23 | -0.20 | 0.66 | -2.91 |
| JP | 1980-1989 | 0.52 | -1.64 | 1.47 | 0.93 | -2.83 |
|  | 1985-1994 | 0.35 | -1.29 | 0.53 | 0.42 | 2.03 |
|  | 1990-1999 | 0.24 | -1.43 | 1.35 | 0.45 | -3.01 |
|  | 1995-2004 | 0.88 | -1.18 | -0.72 | 0.92 | 2.10 |
|  | 2000-2009 | 0.40 | -3.06 | -4.10 | 0.32 | 0.34 |
|  | 2005-2014 | -0.65 | -4.45 | -3.31 | 0.73 | -2.97 |
|  | 2010-2019 | -0.20 | -4.25 | -1.03 | 0.81 | -2.64 |
|  | 2013-2022 | -0.96 | -3.97 | -1.86 | 0.66 | -1.83 |
| SWUS | 1980-1989 | 0.07 | 0.69 | 0.11 | 0.70 | -2.88 |
|  | 1985-1994 | -0.24 | -2.29 | -1.77 | 0.39 | 2.48 |
|  | 1990-1999 | -0.70 | -1.33 | -2.42 | 0.14 | -6.42 |
|  | 1995-2004 | -0.10 | -1.44 | -3.10 | 1.06 | -0.39 |
|  | 2000-2009 | -0.24 | -2.81 | -3.22 | 0.27 | 1.86 |
|  | 2005-2014 | -0.41 | -3.56 | -3.27 | 0.69 | -1.63 |
|  | 2010-2019 | -0.06 | -3.90 | -1.66 | 1.05 | 2.45 |
|  | 2013-2022 | 0.02 | -4.48 | -2.18 | 0.76 | 5.60 |
| CUS | 1980-1989 | 0.55 | -0.20 | -1.45 | 0.74 | -2.16 |
|  | 1985-1994 | -0.79 | -0.14 | -2.04 | -0.24 | 0.84 |
|  | 1990-1999 | 0.71 | -0.46 | -2.56 | 0.98 | -4.99 |
|  | 1995-2004 | -0.44 | -1.84 | -2.75 | 0.38 | 1.08 |
|  | 2000-2009 | -0.61 | -2.96 | -2.59 | -0.08 | -0.03 |
|  | 2005-2014 | -0.68 | -4.05 | -1.88 | 0.34 | -1.75 |
|  | 2010-2019 | -0.58 | -4.34 | -2.05 | -0.12 | -1.53 |
|  | 2013-2022 | -0.20 | -4.73 | -2.68 | 0.73 | 0.31 |
| SEUS | 1980-1989 | -0.02 | -0.23 | -0.59 | 0.13 | -1.71 |
|  | 1985-1994 | -1.08 | -1.67 | -1.94 | 0.07 | 1.30 |
|  | 1990-1999 | 0.52 | -1.11 | -1.99 | 0.41 | -4.90 |
|  | 1995-2004 | -0.84 | -1.25 | -2.82 | 0.10 | 0.90 |
|  | 2000-2009 | -0.56 | -3.48 | -3.44 | 0.11 | 0.72 |
|  | 2005-2014 | -1.11 | -3.89 | -2.79 | 0.10 | -2.63 |
|  | 2010-2019 | -0.51 | -3.85 | -1.41 | 0.10 | -2.10 |
|  | 2013-2022 | -0.14 | -4.52 | -1.92 | 0.48 | -0.03 |
| NEUS | 1980-1989 | 0.04 | -0.25 | -1.37 | 0.53 | -1.30 |
|  | 1985-1994 | -0.33 | -0.35 | -1.69 | 0.39 | -0.55 |
|  | 1990-1999 | 0.57 | -1.57 | -2.79 | 1.15 | -4.56 |
|  | 1995-2004 | -0.53 | -2.51 | -3.28 | 0.78 | 2.95 |
|  | 2000-2009 | -0.37 | -3.61 | -4.45 | 0.77 | -1.79 |
|  | 2005-2014 | -0.51 | -4.52 | -4.59 | 0.49 | -2.91 |
|  | 2010-2019 | -0.59 | -4.40 | -1.89 | 0.25 | -1.09 |
|  | 2013-2022 | -0.41 | -5.26 | -2.42 | 0.96 | 0.89 |
| EU | 1990-1999 | 0.15 | -2.81 | -4.24 | 0.63 | -5.06 |
|  | 1995-2004 | 0.66 | -1.62 | -3.50 | 1.16 | -0.03 |
|  | 2000-2009 | -0.26 | -2.59 | -3.81 | 0.52 | -0.29 |
|  | 2005-2014 | -0.30 | -4.14 | -3.37 | 0.23 | -2.40 |
|  | 2010-2019 | 0.27 | -3.04 | -1.14 | 1.14 | 0.14 |
|  | 2013-2022 | 0.00 | -3.49 | -0.89 | 1.11 | 1.15 |

^a^ Decadal warm-season mean temperature at each grid is first removed before deriving the temperature trends.

**Table S5.** HCHO/NO_2_ ratio averaged over 11 regions across the Northern Hemisphere.

| Warm season | | | | | | | | |
| --- | --- | --- | --- | --- | --- | --- | --- | --- |
| Region | 1980-1989 | 1985-1994 | 1990-1999 | 1995-2004 | 2000-2009 | 2005-2014 | 2010-2014 | 2013-2014 |
| NCP | / | / | / | / | / | / | / | 0.16 |
| YRD | / | / | / | / | / | / | / | 0.22 |
| PRD | / | / | / | / | / | / | / | 0.23 |
| SCB | / | / | / | / | / | / | / | 0.19 |
| Kr | 0.42 | 0.34 | 0.28 | 0.26 | 0.29 | 0.40 | 0.46 | 0.48 |
| JP | 0.15 | 0.18 | 0.22 | 0.27 | 0.32 | 0.37 | 0.40 | 0.42 |
| SWUS | 1.31 | 1.48 | 1.39 | 1.31 | 1.31 | 1.19 | 1.09 | 1.06 |
| CUS | 1.63 | 1.76 | 3.21 | 3.76 | 3.77 | 3.40 | 2.97 | 2.86 |
| SEUS | 3.76 | 4.20 | 4.58 | 5.04 | 4.92 | 4.58 | 4.16 | 4.15 |
| NEUS | 0.47 | 0.47 | 0.49 | 0.48 | 0.51 | 0.66 | 0.69 | 0.77 |
| EU | 0.18 | 0.18 | 0.19 | 0.30 | 0.42 | 0.49 | 0.57 | 0.62 |
| Cold season | | | | | | | | |
| region | 1980-1989 | 1985-1994 | 1990-1999 | 1995-2004 | 2000-2009 | 2005-2014 | 2010-2014 | 2013-2014 |
| NCP | / | / | / | / | / | / | / | 18.7 |
| YRD | / | / | / | / | / | / | / | 15.5 |
| PRD | / | / | / | / | / | / | / | 8.1 |
| SCB | / | / | / | / | / | / | / | 9.6 |
| Kr | 0.09 | 0.07 | 0.06 | 0.06 | 0.07 | 0.08 | 0.09 | 0.10 |
| JP | 0.04 | 0.05 | 0.06 | 0.07 | 0.08 | 0.09 | 0.10 | 0.11 |
| SWUS | 0.72 | 0.79 | 0.78 | 0.73 | 0.71 | 0.66 | 0.61 | 0.61 |
| CUS | 1.01 | 1.11 | 2.15 | 2.53 | 2.49 | 2.20 | 1.86 | 1.88 |
| SEUS | 2.59 | 2.91 | 3.29 | 3.57 | 3.44 | 3.13 | 2.77 | 2.83 |
| NEUS | 0.18 | 0.17 | 0.17 | 0.17 | 0.18 | 0.22 | 0.24 | 0.26 |
| EU | 0.07 | 0.06 | 0.06 | 0.10 | 0.15 | 0.18 | 0.22 | 0.25 |

**Table S6.** Configurations of model simulations.

| Simulation ID | Region | Anthropogenic emissions | Meteorology | Biomass burning |
| --- | --- | --- | --- | --- |
| 1 | China | 2013 | 2013 | 2013 |
| 2 |  | 2020 | 2013 | 2013 |
| 3 |  | 2020 | 2022 | 2022 |
| 4 | South Korea | 2005 | 2005 | 2005 |
| 5 |  | 2014 | 2005 | 2005 |
| 6 |  | 2013 | 2013 | 2013 |
| 7 |  | 2020 | 2013 | 2013 |
| 8 |  | 2013_Other  2020_NMVOCs | 2013 | 2013 |
| 9 | Japan | 2013 | 2013 | 2013 |
| 10 |  | 2020 | 2013 | 2013 |
| 11 |  | 2013_Other  2020_NMVOCs | 2013 | 2013 |
| 12 | Europe | 2013 | 2013 | 2013 |
| 13 |  | 2013 | 2022 | 2013 |
| 14 |  | 2013 | 2013 | 2022 |
| 15 | US | 2013 | 2013 | 2013 |
| 16 |  | 2013 | 2022 | 2013 |
| 17 |  | 2013 | 2013 | 2022 |


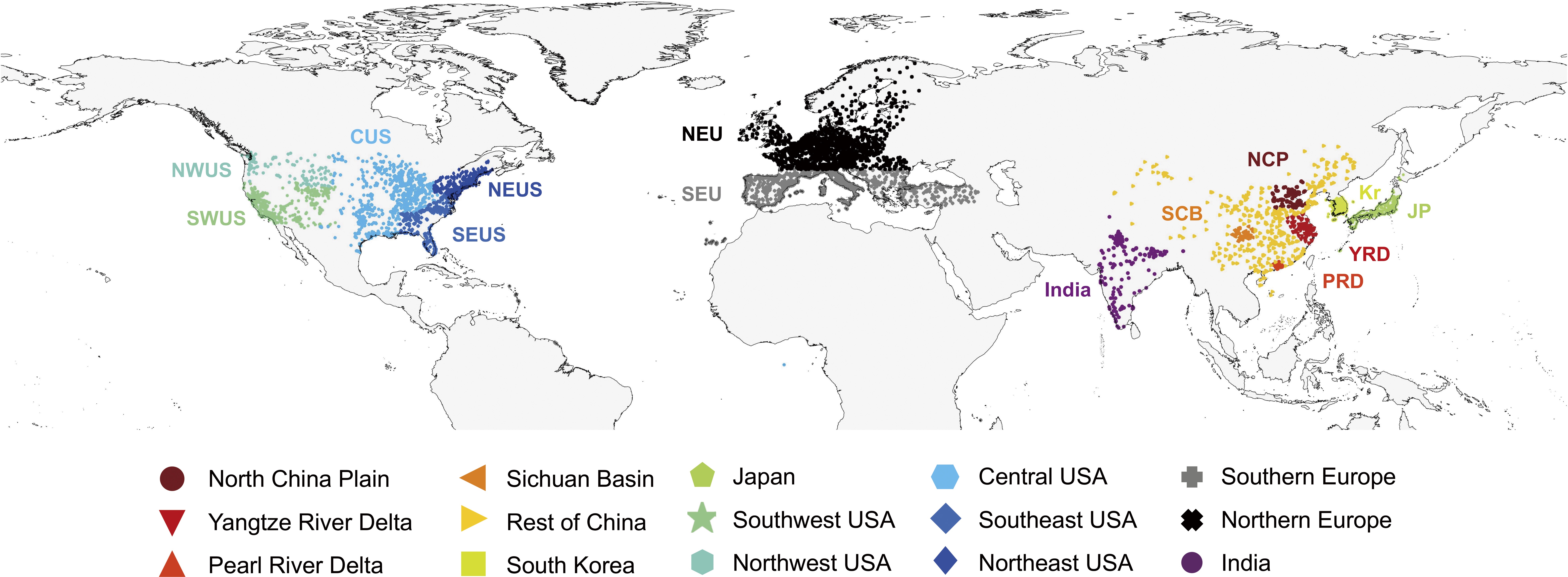


Figure S1. Locations of continuous ozone observations over 2013–2022 used in this study. These sites are from the North China Plain (NCP) city cluster, the Yangtze River Delta (YRD) city cluster, the Pearl River Delta (PRD) city cluster, Sichuan Basin (SCB), South Korea (Kr), Japan (JP), Southwest USA (SWUS), Northwest USA (NWUS), Central USA (CUS), Southeast USA (SEUS), Northeast USA (NEUS), Southern Europe (SEU), Northern Europe (NEU), and India.


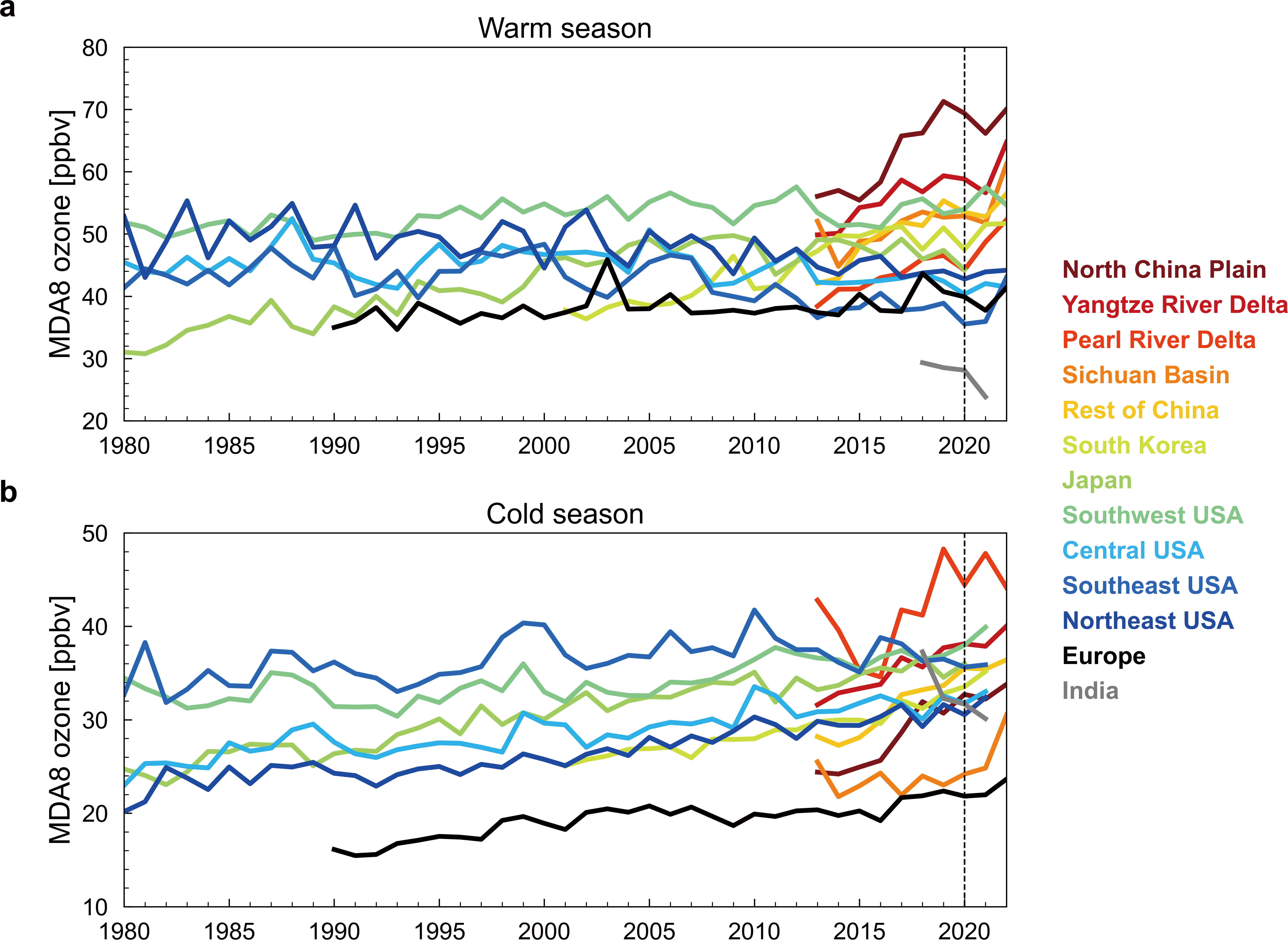


Figure S2. Evolution of surface MDA8 ozone levels in thirteen representative regions in the (a) warm season and (b) cold season from 1980 to 2022. Sites are consistent with Figure S7, with available monthly records for more than 60% considered in all decades. NWUS is excluded here due to the absence of stations meeting the criteria.


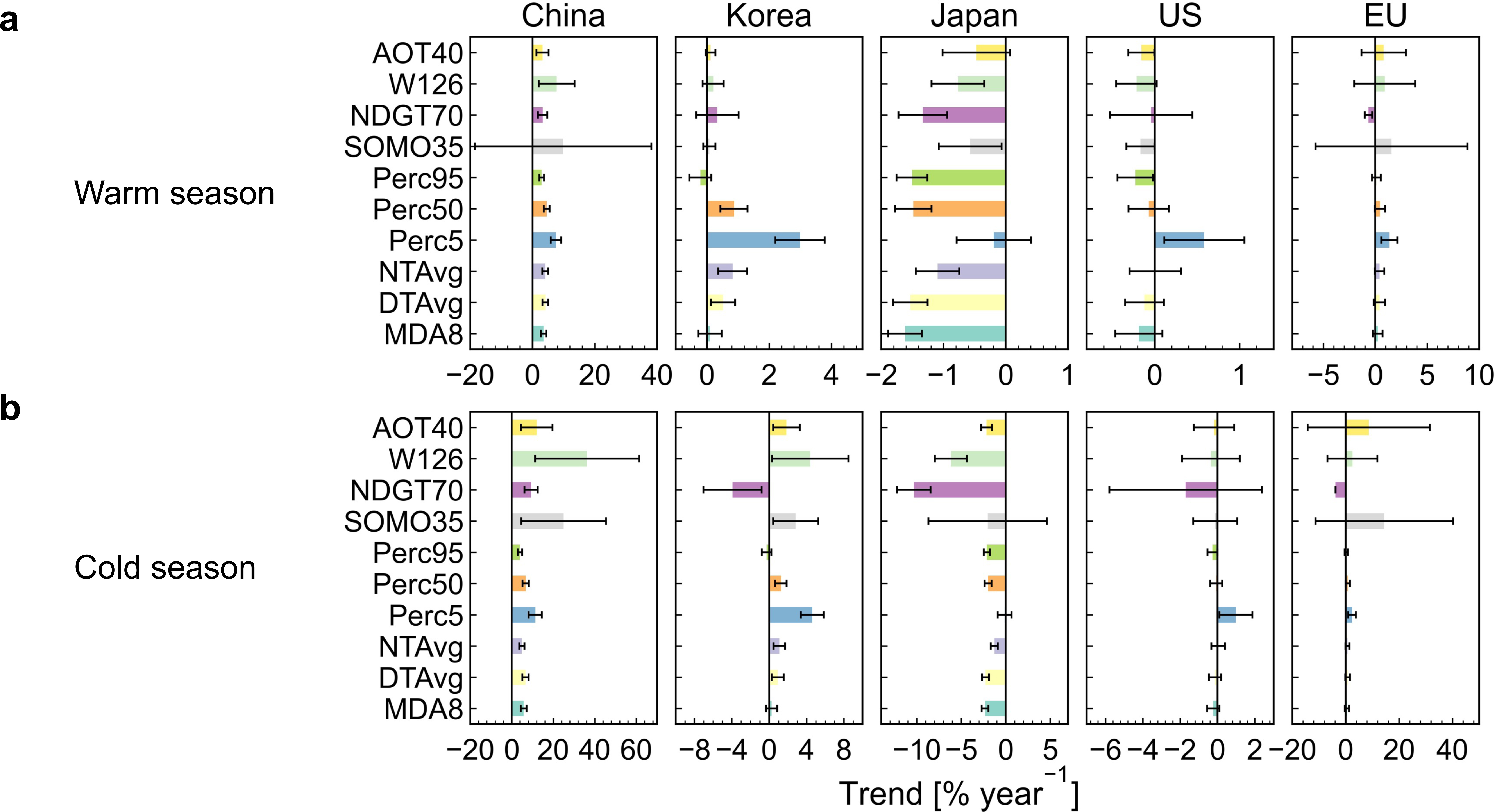


Figure S3. Percentages of annual trend (mean ± 25% standard deviation) for different ozone metrics for the (a) warm and (b) cold seasons in 2013–2022 at the five national ozone monitoring sites.


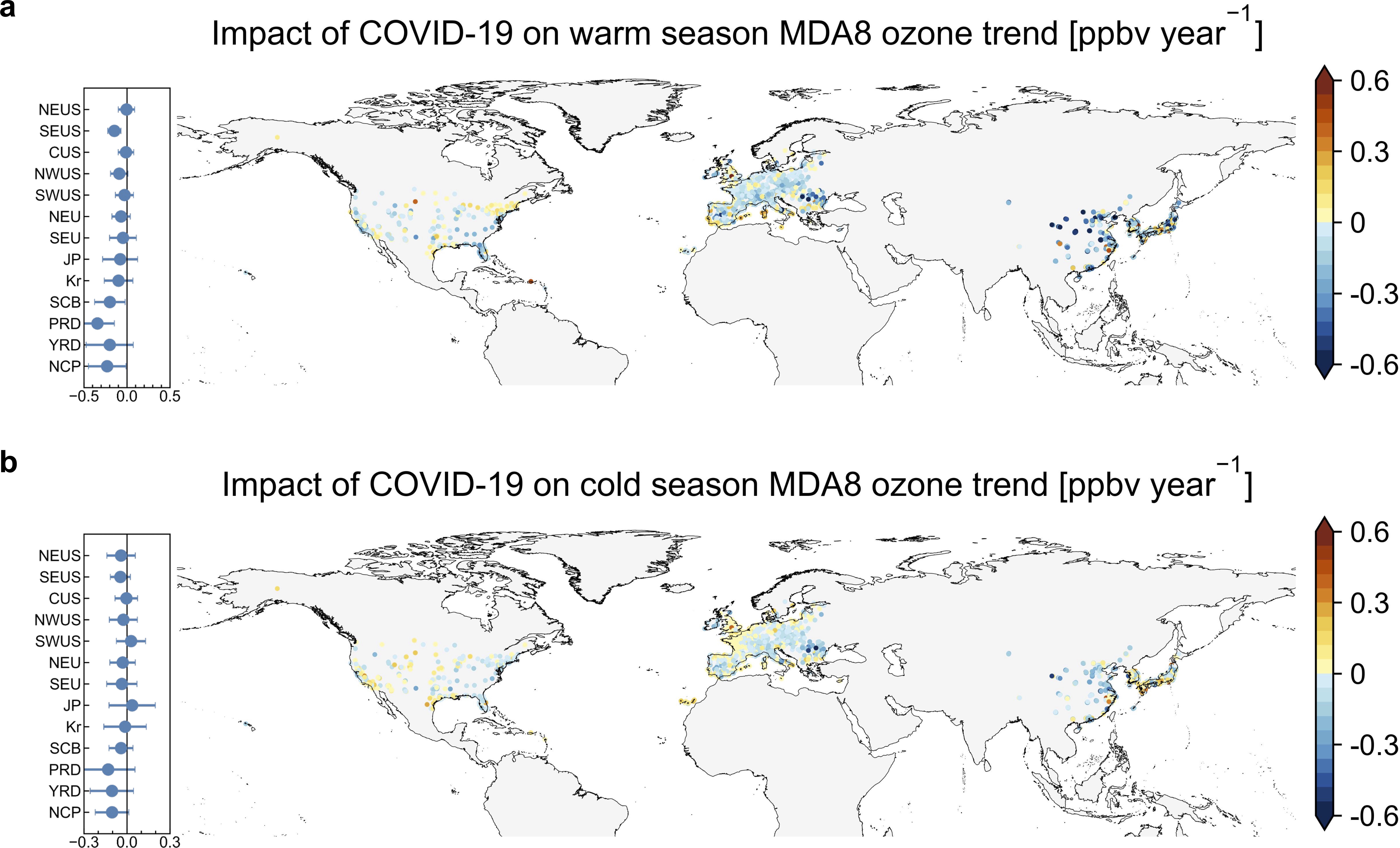


Figure S4. Impact of COVID-19 on MDA8 ozone for the (a) warm and (b) cold seasons at ozone monitoring sites in five countries, i.e., the difference in the 2013–2022 ozone trend in the presence vs absence of the 2020 data. The inset shows the average change in the MDA8 ozone trend in each region. The horizon bars indicate the standard deviation of change in the MDA8 ozone trend across all sites within the region.


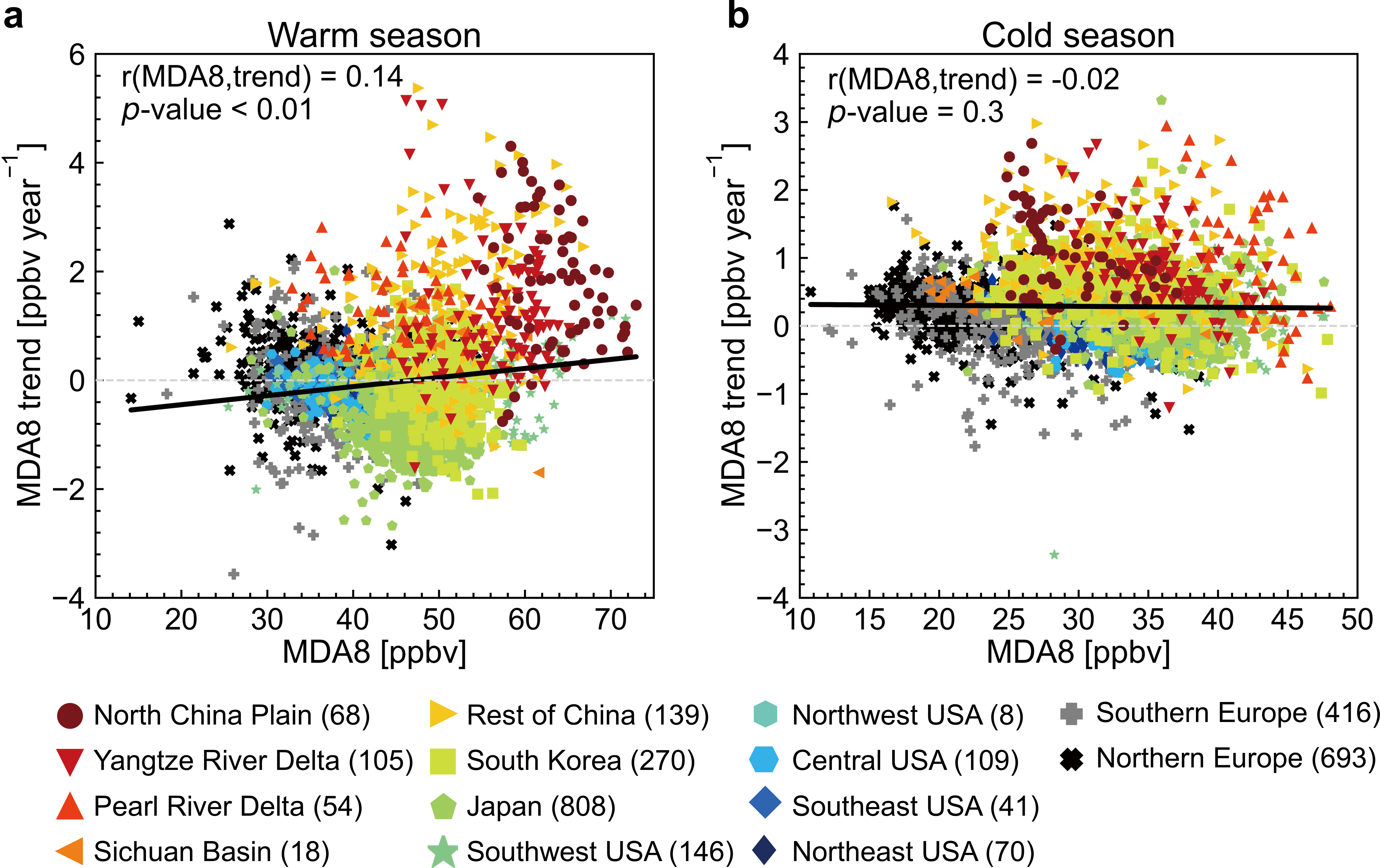


Figure S5. Same as Figure 2(a), but for individual sites for both warm and cold season.


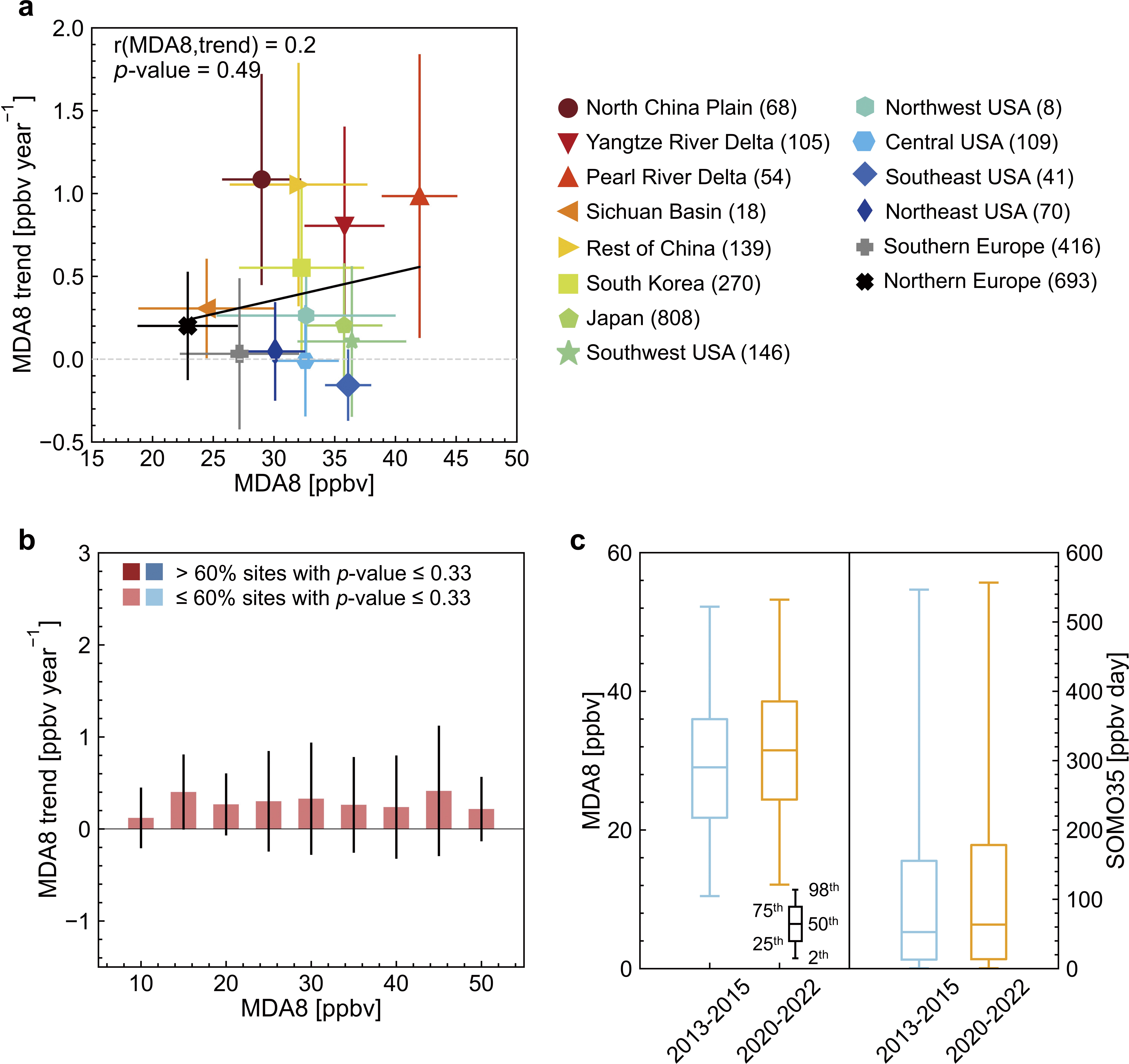


Figure S6. Same as Figure 2 but for the cold season.


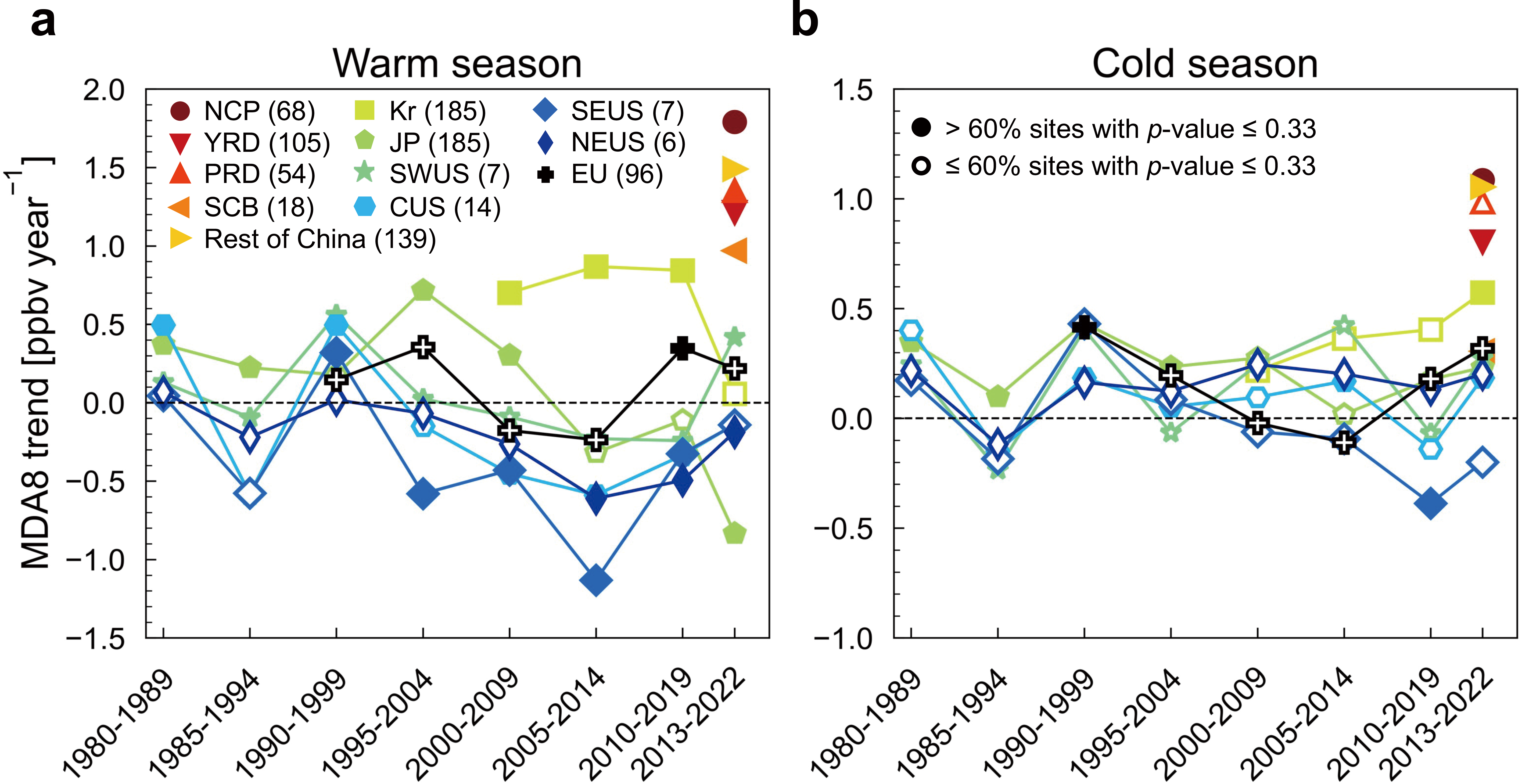


Figure S7. Same as Figure 3, (a) and (b) but using continuous observation sites with at least 60% of available monthly records in all decades.


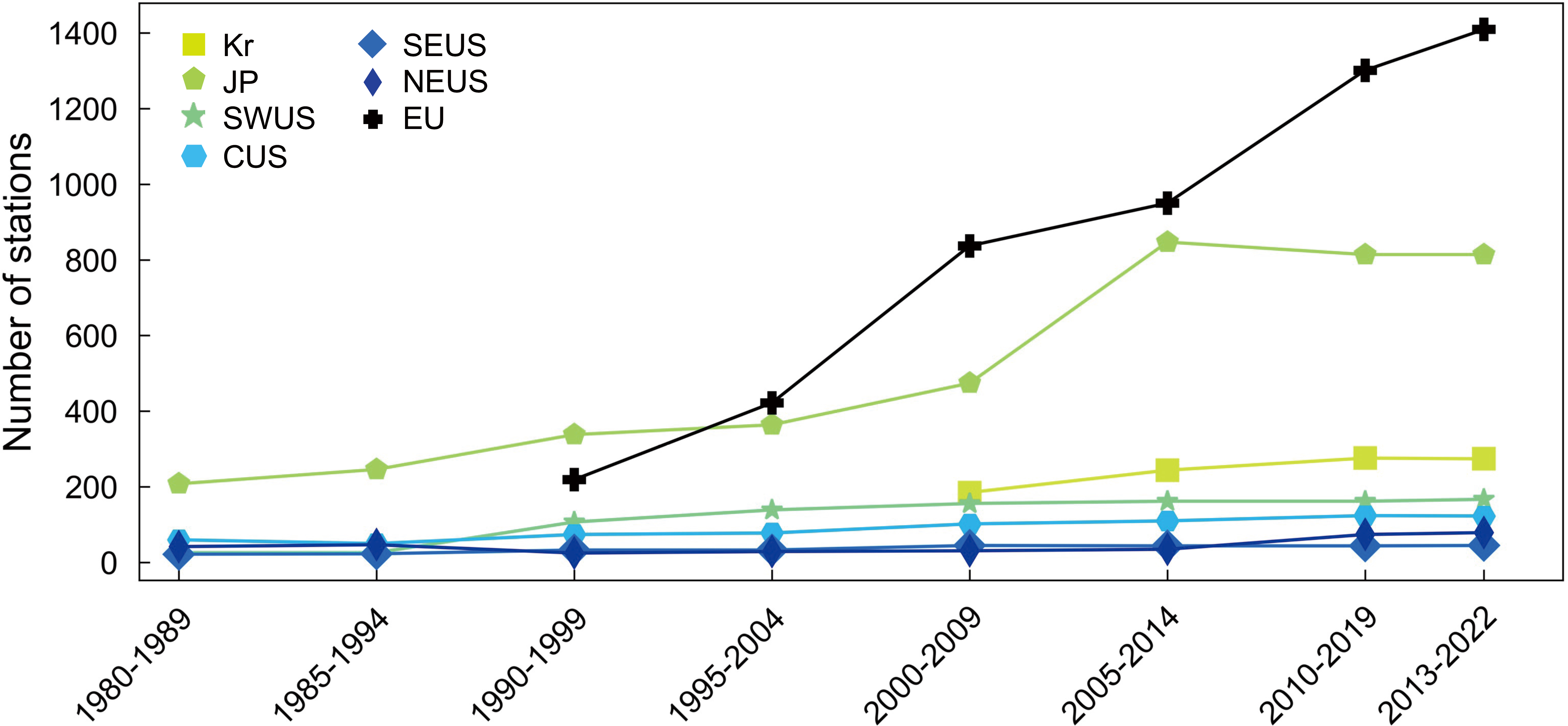


Figure S8. Evolution of the number of sites used to derive the mean MDA8 ozone trends in different decades and regions in Figure 3.


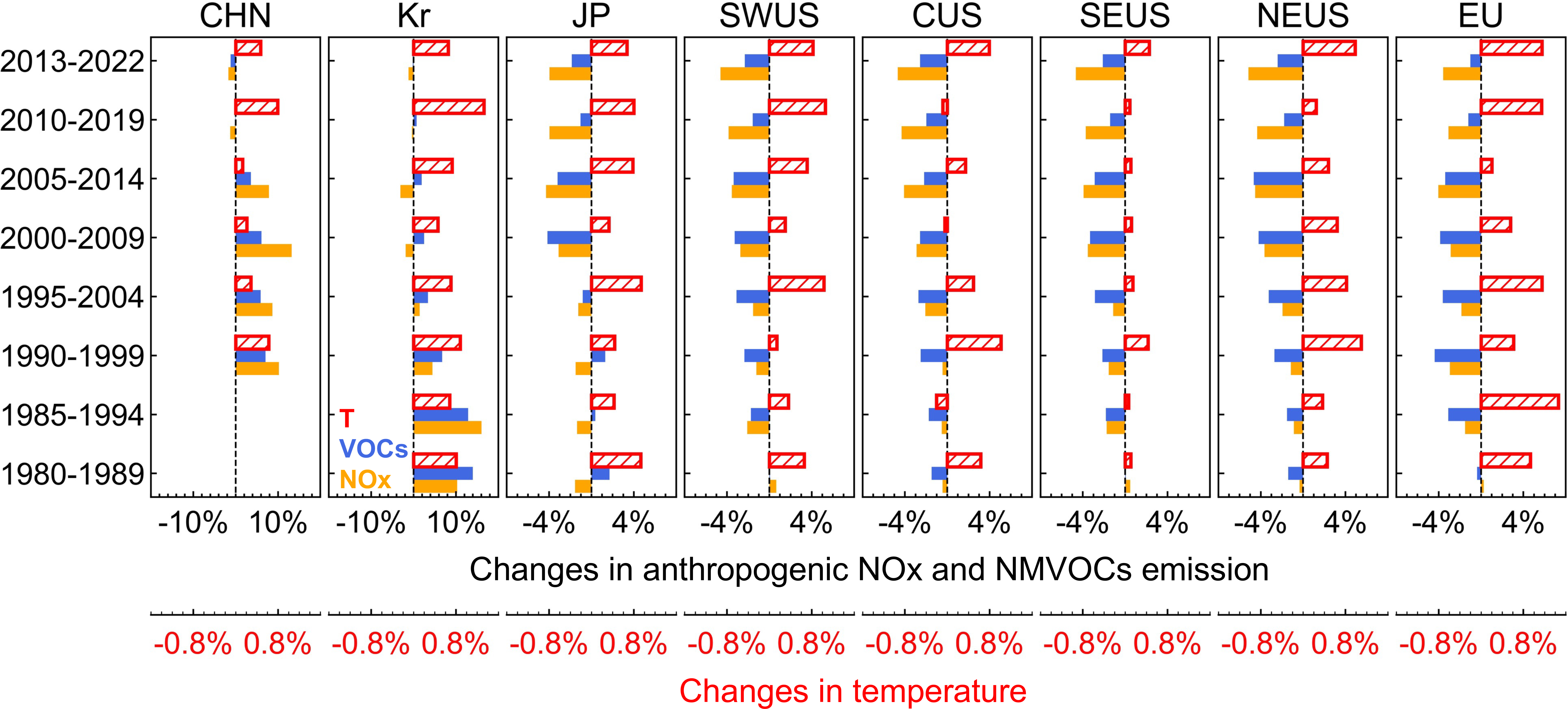


Figure S9. The magnitude of changes in daytime average temperature (red bars; from MERRA2 reanalysis data), anthropogenic NO*_x_* (orange bars), and NMVOCs emissions (blue bars) from the Community Emissions Data System (CEDS) emissions over the different decade periods. The changes in all the elements are subsequently mapped to the corresponding stations and then averaged across distinct time windows. The changes in anthropogenic emissions from 2013–2022 in the graph represent the period of 2013–2020.


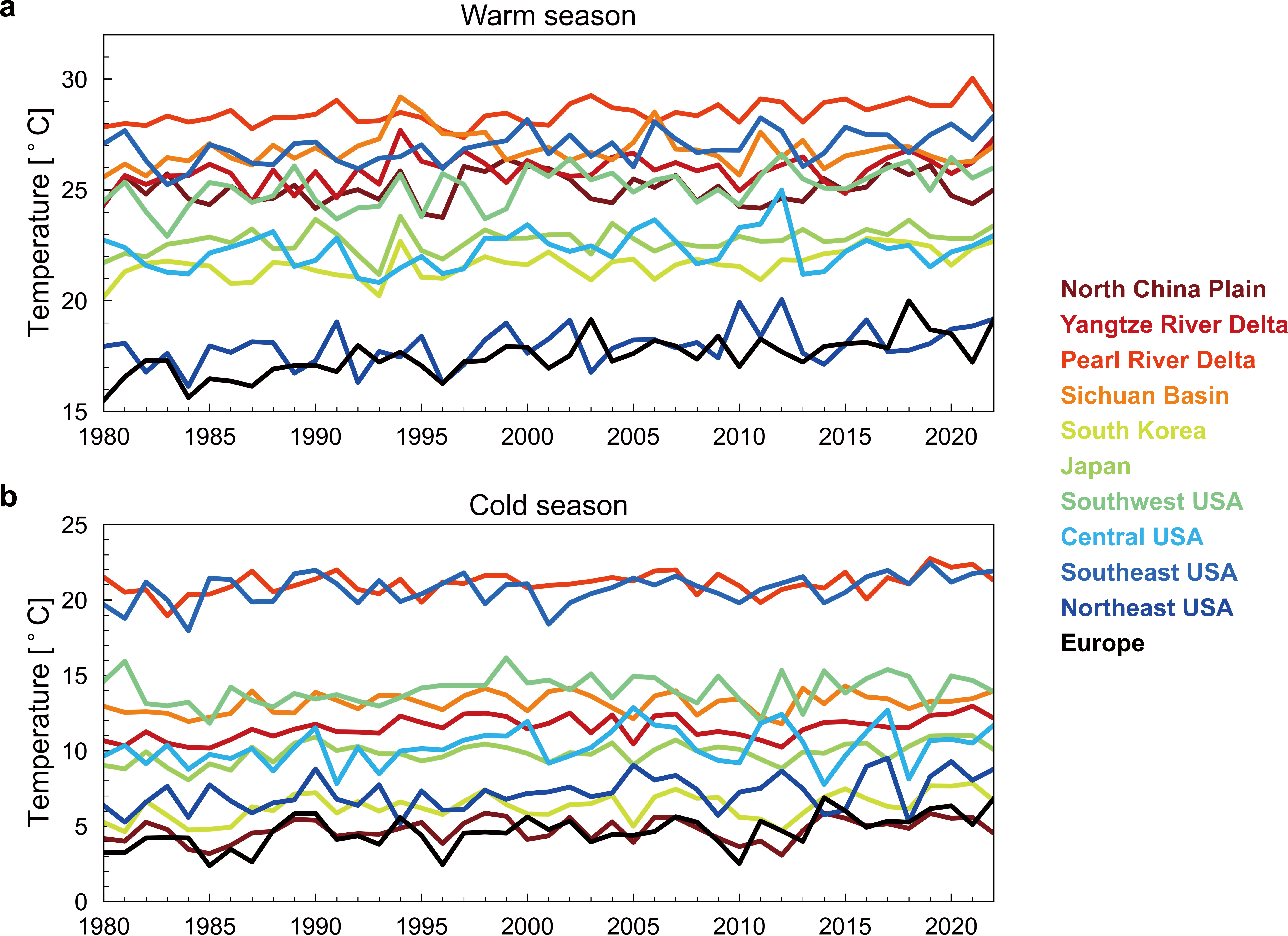


Figure S10. Evolution of daytime average temperature in eleven representative regions in the (a) warm season and (b) cold season from 1980 to 2022. The data are from Modern-Era Retrospective analysis for Research and Application version 2 (MERRA-2) reanalysis meteorological data. Sites are consistent with Figure S7, with available monthly records for more than 60% considered in all decades. The grid data are mapped to the corresponding stations and then averaged over the time windows.


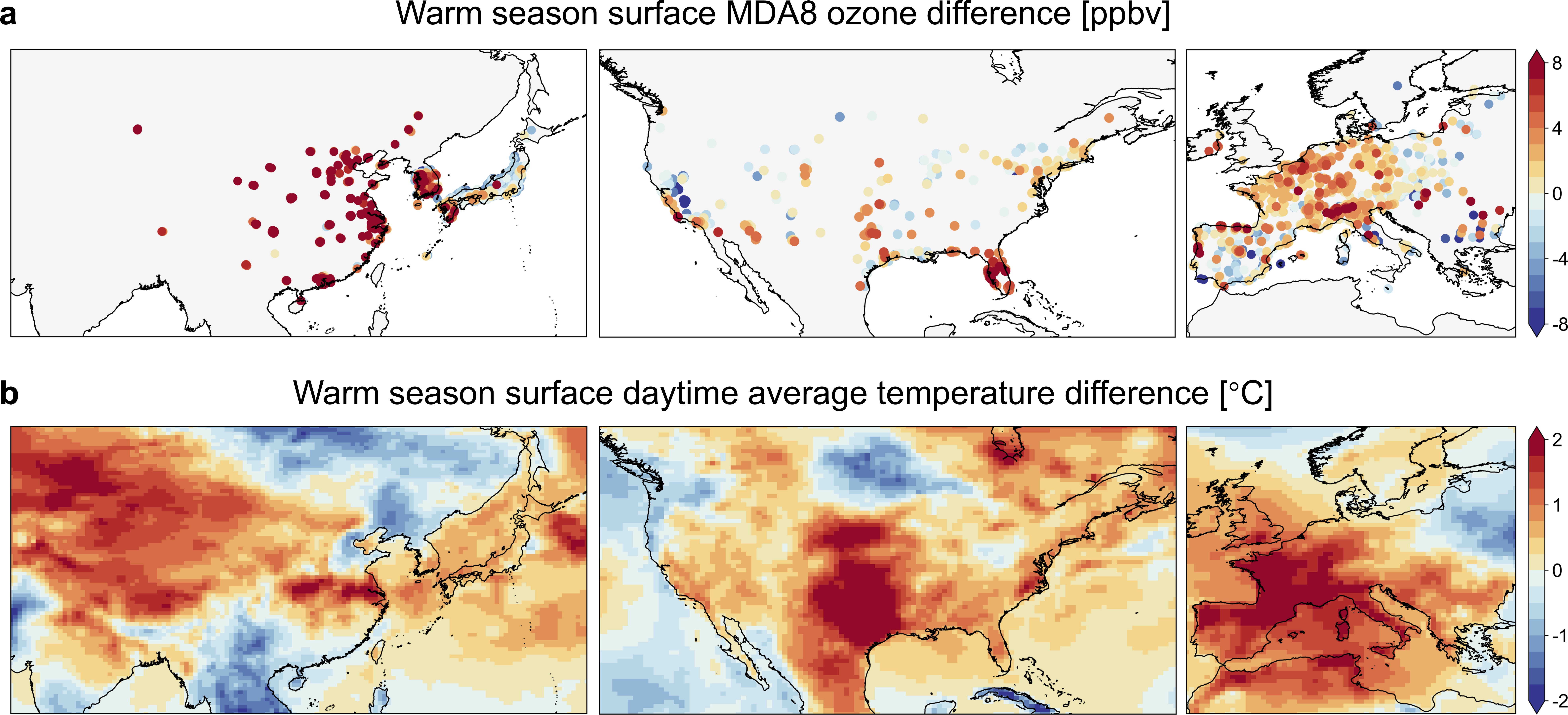


Figure S11. Spatial consistency between warm-season MDA8 ozone and daytime average temperature anomalies. Panel (a) shows MDA8 ozone concentration anomalies in 2022 relative to 2013–2021. Panel (b) is the same as Panel (a) but for daytime average temperature.


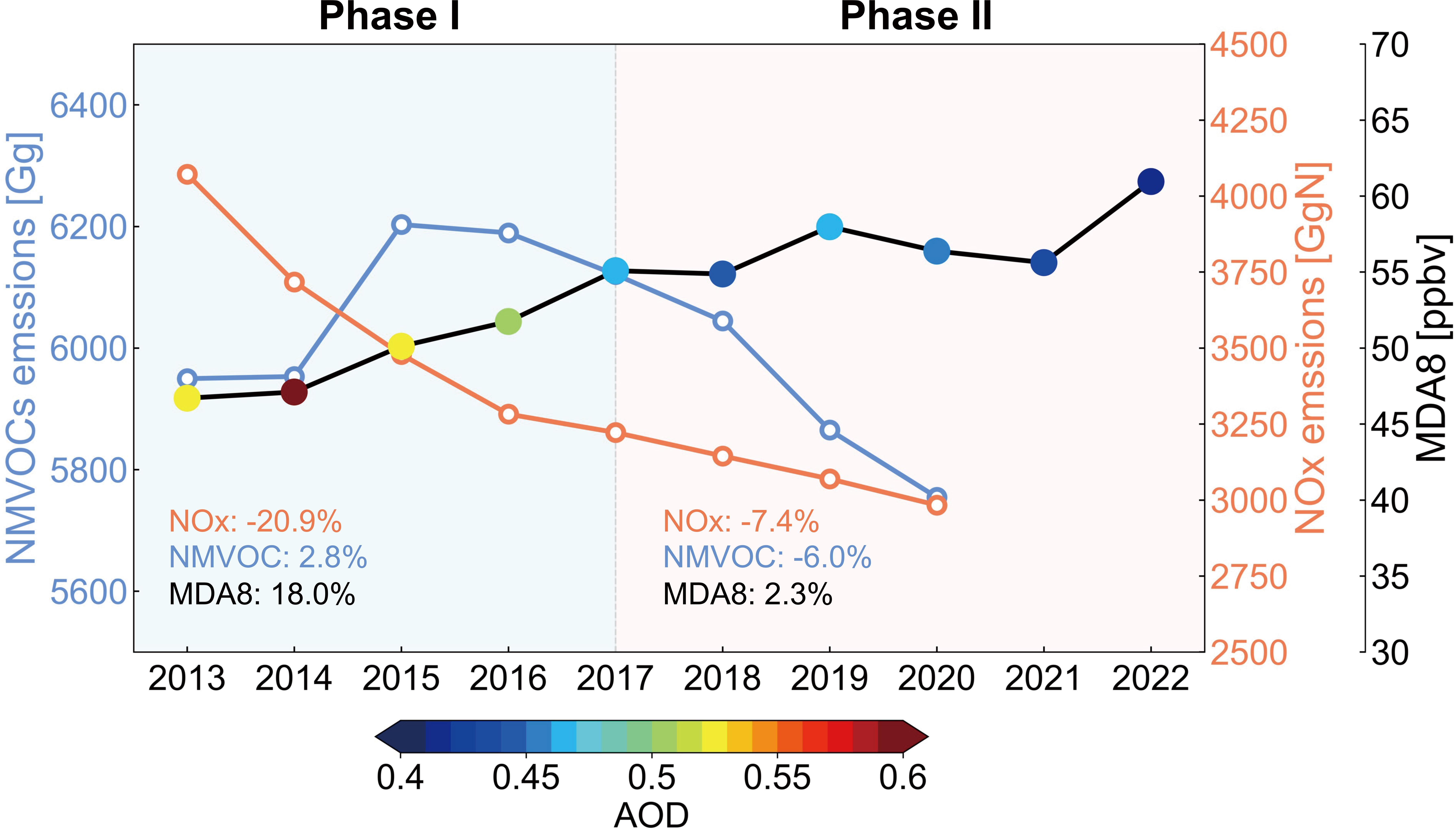


**Figure S12.** Evolution of MDA8 ozone, NMVOCs, NO*_x_* emissions, and AOD in China in the warm season from 2013 to 2022. The inset shows the magnitude of changes in MDA8 ozone, NMVOCs, and NO*_x_* emissions during the two phases.


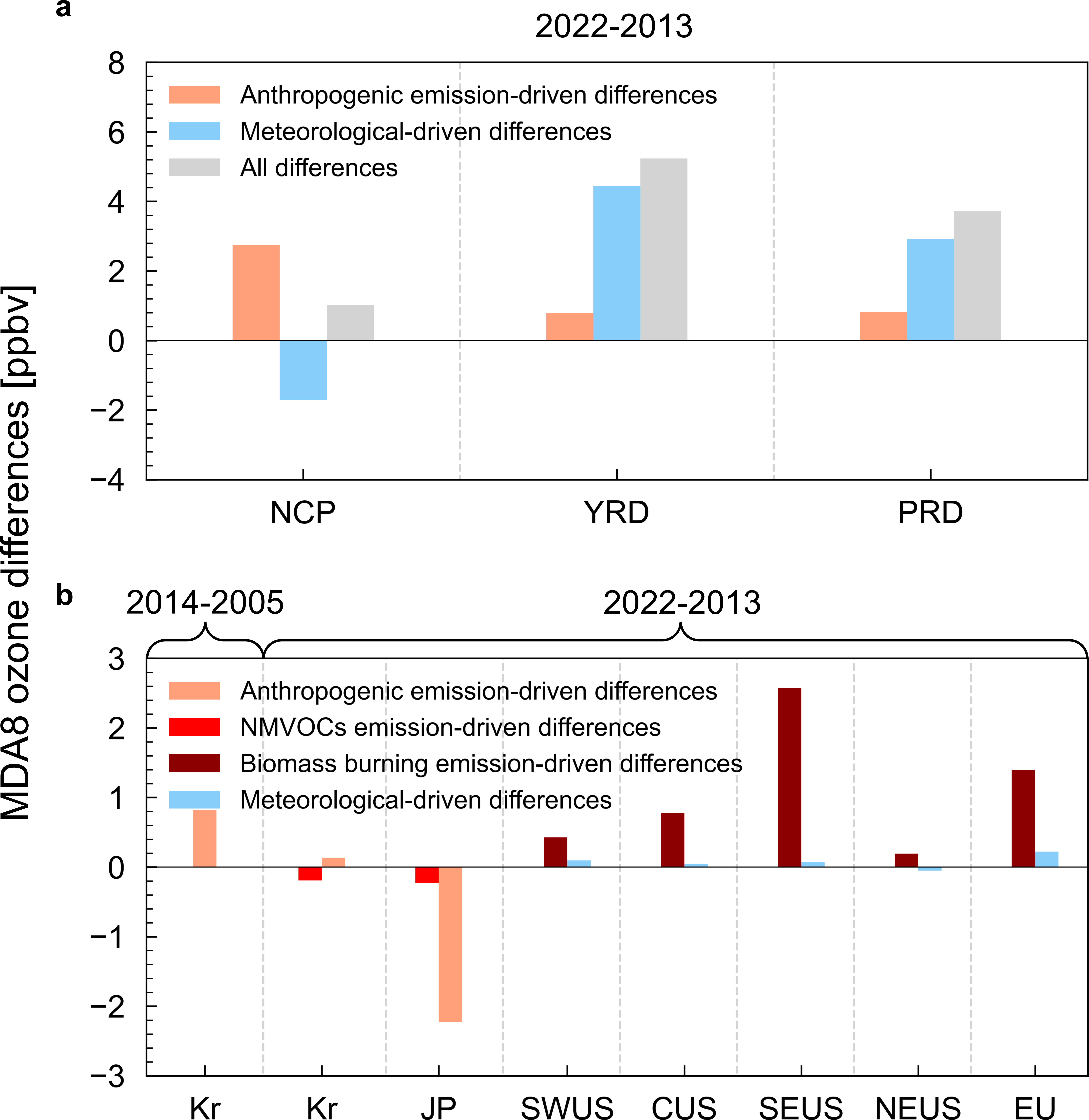


**Figure S13.** Contribution of anthropogenic emissions, biomass burning emissions, and weather conditions to ozone changes in different regions and periods, estimated from the GEOS-Chem simulation. Panel (**a**) shows the ozone differences between 2022 and 2013 for three regions in China, including total, anthropogenic emissions, and meteorological-driven differences. Panel (**b**) presents the attribution of ozone differences for South Korea, Japan, Europe, and four regions in the US. The contribution of anthropogenic emissions to the change in ozone in Korea in 2014 relative to 2005 has been quantified. For South Korea and Japan, the analysis shows the contribution of changes in NMVOCs emissions and total anthropogenic emissions from 2022 relative to 2013. In Europe and the US, meteorology and biomass burning were altered to elucidate their respective contributions on ozone difference between 2022 and 2013.


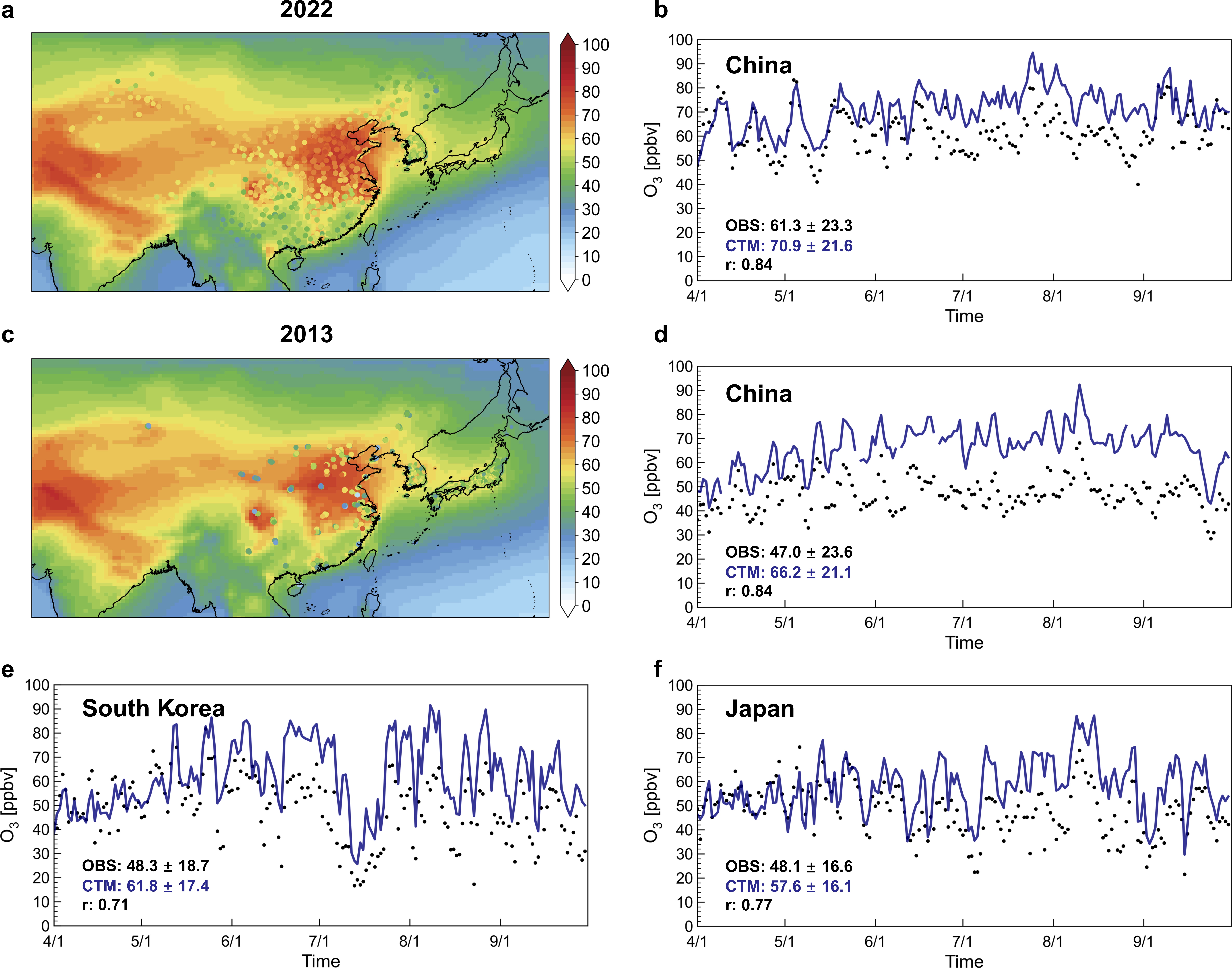


**Figure S14.** Observed (dots) and simulated (shaded) warm-season surface MDA8 ozone for (**a**) 2022 and (**c**) 2013. Time-series of observed (blue line) and simulated (black dots) warm-season surface ozone over (**b**) the China for 2022, (**d**) the China for 2013, (**e**) the South Korea for 2013, and (**f**) the Japan for 2013. Mean values ± standard deviation and the spatial correlation coefficients (r) are shown in the inset.


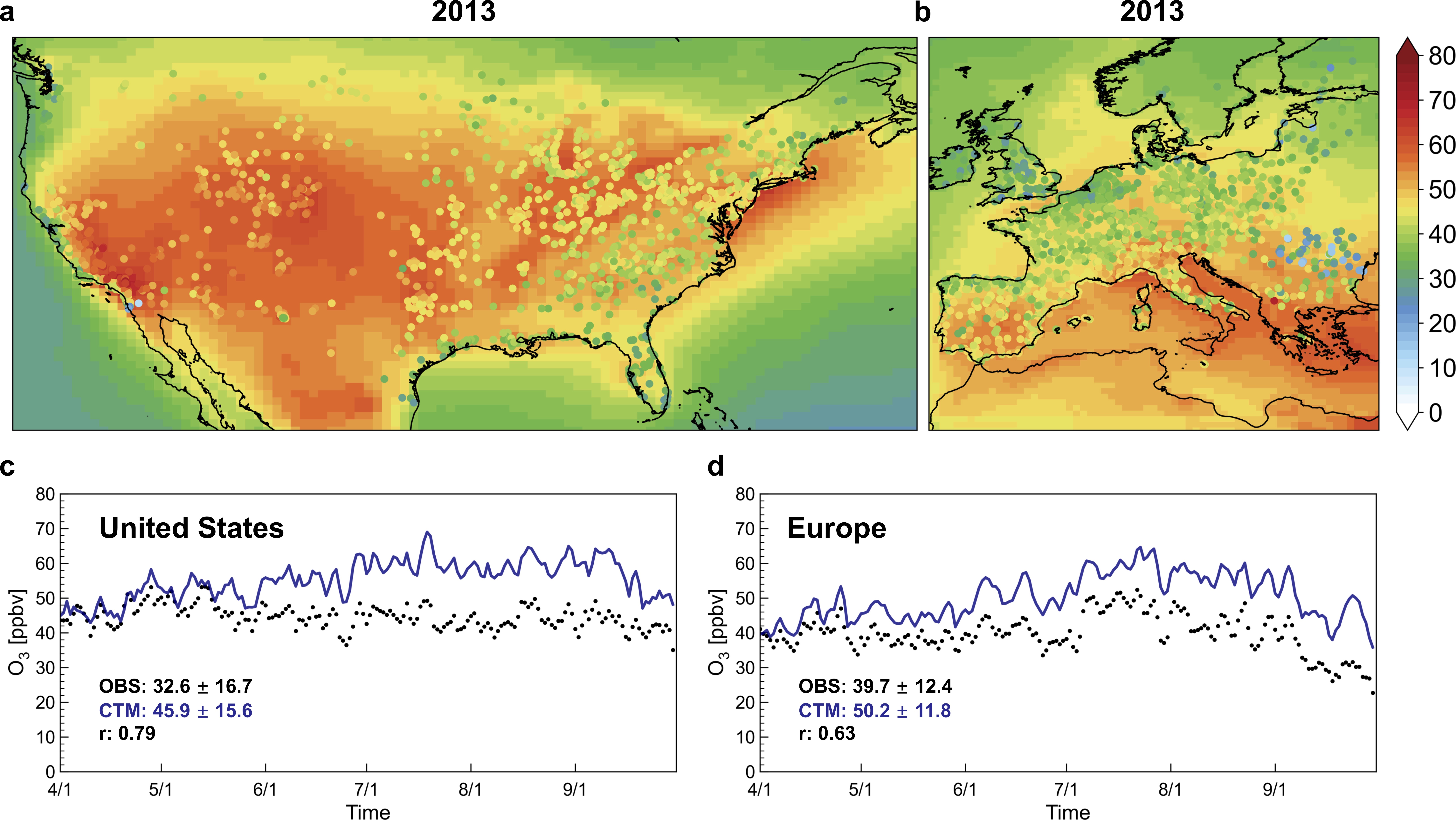


**Figure S15.** Observed (dots) and simulated (shaded) warm-season surface MDA8 ozone over (**a**) the US and (**b**) the Europe for 2013. Time-series of observed (blue line) and simulated (black dots) warm-season surface ozone over (**c**) the US and (**d**) the Europe for 2013. Mean values ± standard deviation and the spatial correlation coefficients (r) are shown in the inset.

References

1. Wang H, Wang H, Lu X *et al.* Increased night-time oxidation over China despite widespread decrease across the globe. *Nat Geosci* 2023; **16**: 217–23.

2. Lefohn AS, Malley CS, Smith L *et al.* Tropospheric ozone assessment report: Global ozone metrics for climate change, human health, and crop/ecosystem research. *Elem Sci Anth* 2018; **6**: 27.

3. Chang K-L, Schultz MG, Lan X *et al.* Trend detection of atmospheric time series: Incorporating appropriate uncertainty estimates and handling extreme events. *Elem Sci Anth* 2021; **9**: 00035.

4. Lu X, Zhang L, Wang X *et al.* Rapid increases in warm-season surface ozone and resulting health impact in China since 2013. *Environ Sci Technol Lett* 2020; **7**: 240-7.

5. Chang K-L, Schultz MG, Koren G *et al.* Guidance note on best statistical practices for TOAR analyses. *arXiv preprint arXiv:230414236* 2023.

6. Theil H. A rank-invariant method of linear and polynomial regression analysis, 1-2; confidence regions for the parameters of linear regression equations in two, three and more variables. *Indagationes Mathematicae* 1950; **1**.

7. Sen PK. Estimates of the Regression Coefficient Based on Kendall's Tau. *J Am Stat Assoc* 1968; **63**: 1379-89.

8. Jin X, Holloway T. Spatial and temporal variability of ozone sensitivity over China observed from the Ozone Monitoring Instrument. *J Geophys Res: Atmos* 2015; **120**: 7229-46.

9. Bey I, Jacob DJ, Yantosca RM *et al.* Global modeling of tropospheric chemistry with assimilated meteorology: Model description and evaluation. *J Geophys Res: Atmos* 2001; **106**: 23073-95.

10. Wang H, Lu X, Jacob DJ *et al.* Global tropospheric ozone trends, attributions, and radiative impacts in 1995–2017: an integrated analysis using aircraft (IAGOS) observations, ozonesonde, and multi-decadal chemical model simulations. *Atmos Chem Phys* 2022; **22**: 13753-82.

11. Zheng B, Tong D, Li M *et al.* Trends in China's anthropogenic emissions since 2010 as the consequence of clean air actions. *Atmos Chem Phys* 2018; **18**: 14095-111.
